# Cinnamtannin B1 attenuates rosacea-like signs via inhibition of pro-inflammatory cytokine production and down-regulation of the MAPK pathway

**DOI:** 10.7717/peerj.10548

**Published:** 2020-12-21

**Authors:** Hung-Lin Kan, Chia-Chi Wang, Yin-Hua Cheng, Chi-Lung Yang, Hsun-Shuo Chang, Ih-Sheng Chen, Ying-Chi Lin

**Affiliations:** 1 Doctoral Degree Program in Toxicology, College of Pharmacy, Kaohsiung Medical University, Kaohsiung, Taiwan; 2 Department and Graduate Institute of Veterinary Medicine, School of Veterinary Medicine, National Taiwan University, Taipei, Taiwan; 3 School of Pharmacy, College of Pharmacy, Kaohsiung Medical University, Kaohsiung, Taiwan

**Keywords:** Rosacea, Cinnamtannin B1, Anti-inflammation, MAPK

## Abstract

**Background:**

Rosacea is a common inflammatory disease of facial skin. Dysregulation of innate immunity with enhanced inflammation and increased abundance of LL-37 at the epidermal site is a characteristic feature of rosacea. Cinnamtannin B1 (CB1) is a condensed tannin with anti-inflammatory and anti-microbial activities. The aims of the study were to evaluate the potential of CB1 as a therapy for rosacea and to characterize the potential mechanisms of action.

**Methods:**

We intraperitoneally administered 20 mg/kg CB1 once daily for 2 days into the LL-37-induced mouse model of rosacea. The effects of CB1 in vivo were evaluated by the observations of lesions, histology, immunohistochemistry, and the transcription and translation of pro-inflammatory cytokines and chemokines. Human keratinocyte HaCaT and monocyte THP-1 were used to characterize the effects of CB1 on LL-37-induced inflammation in vitro. The changes in pro-inflammatory chemokine interleukin-8 (IL-8) were quantitated by enzyme-linked immunosorbent assay (ELISA), and the expressions of genes involved were determined by Western blotting.

**Results:**

CB1 attenuated local redness, inflammation, and neutrophil recruitment in the mouse model of rosacea in vivo. CB1 suppressed myeloperoxidase (MPO) and macrophage inflammatory protein 2 (MIP-2) production, a functional homolog of interleukin-8 (IL-8), at the lesions. In vitro experiments confirmed that CB1 reversed the LL-37-induced IL-8 production in human keratinocytes HaCaT and monocyte THP-1 cells. CB1 inhibited IL-8 production through downregulating the phosphorylation of extracellular signal-regulated kinase (ERK) in the mitogen-activated protein kinase (MAPK) pathway.

**Conclusion:**

CB1 attenuated LL-37-induced inflammation, specifically IL-8 production, through inhibiting the phosphorylation of ERK. CB1 has potential as a treatment for rosacea.

## Introduction

Rosacea is a chronic skin inflammatory disease common in Caucasians, which is estimated to affect over 15 million adults in the USA alone (Bickers et al., 2006). The symptoms of rosacea include facial redness, transient or persistent erythema, superficial dilated blood vessels, papules, and even thickening sebaceous skin on the nose (Crawford, Pelle & James, 2004). The disease is most prevalent in middle-aged adults and females (Spoendlin et al., 2012) and has been associated with psychosocial distress of the patients. Environmental stimuli, such as ultraviolet radiation, microbes, and chemical irritants, have been known to trigger rosacea.

The pathogenesis of rosacea is not fully understood yet. Over-activation of the innate immune system in response to environmental stimuli such as UV and microbes is known to play important roles (Yamasaki & Gallo, 2009). An enhanced amount of anti-microbial peptide cathelicidin, observed in rosacea regions of the patients, has also been linked to the production of reactive oxygen species (ROS) and inflammatory cytokines and chemokines at the sites. Although cathelicidin is not the singular factor of rosacea causation and the peptide itself is known to mediate both pro- and anti-inflammatory effects based on concentration, cell type, and kinetics of response in different models of inflammation (Yang et al., 2020a). Intradermal injection of LL-37, a cleavage active product of cathelicidin, was shown to reproduce rosacea-like redness and neutrophil recruitment in the animal model (Yamasaki et al., 2007).

Topical and/or systemic anti-microbial agents including azelaic acid, retinoids, metronidazole, tetracyclines, and ivermectin (Pelle, Crawford & James, 2004; Jones, 2009; Fallen & Gooderham, 2012; Cardwell et al., 2016) have been shown to relieve rosacea symptoms clinically. All the above-mentioned agents are also known to have antioxidant effect (Yamasaki & Gallo, 2009). Concerning the adverse effects associated with these agents and antibiotic-resistance issues, botanical therapies may be good alternatives for the patients (Emer, Waldorf & Berson, 2011; Fisk et al., 2015).

Cinnamtannin B1 (CB1) is a condensed tannin with anti-inflammatory and anti-microbial activities. CB1 could reduce the release of reactive oxygen species (ROS) (Gonzalez et al., 2012), inhibit neutrophils activation (Yang et al., 2020b), as well as inhibit the activity of cyclooxygenase-2 (COX-2) (Killday et al., 2011). Our group and others also observed that CB1 has some activity against the growth of *Bacillus subtilis*, *Staphylococcus aureus,* and *Propionibacterium acnes* (Idowu et al., 2010)*.*

Given the anti-oxidant, anti-inflammatory, and anti-microbial activities of CB1, we aimed to investigate the potential of CB1 as a therapy for rosacea. In this study, we demonstrated the anti-inflammatory activities of CB1 in vivo and in vitro in LL-37-induced inflammation and further characterized the cellular mechanisms involved.

## Materials and Methods

### Preparation of Cinnamtannin B1 (CB1)

The CB1 used in this study was isolated from the stem of Formosan *Cinnamomum validinerve*, which was collected at Mudan mountain at Pingtung County, Taiwan. The plant sample was identified by one of the authors, Prof. Ih-Sheng Chen, and the voucher specimen was deposited in the Herbarium of the College of Pharmacy, Kaohsiung Medical University, Kaohsiung, Taiwan. The separation and isolation of the CB1 has been reported (Yang et al., 2020b). Briefly, the stem was dried and extracted with MeOH. The extract was then partitioned with EtOAc: H_2_O (1:1) solvent system and then separated by column chromatography. The purity of CB1 in this preparation was over 90% based on ^1^H & ^13^C NMR analysis.

### Reagents and chemicals

The cleaved human cathelicidin peptide LL-37 (LLGDFFRKSKEKIGKEFKRIVQRIKDFLRNLVPRTES) was customarily synthesized with 95% purity and without any modifications by Genemed Synthesis Inc. (South San Francisco, CA). The chemicals used in the animal model were obtained from Sigma (St Louis, MO, USA). Reagents for reverse transcription-polymerase chain reactions (RT-PCR) were purchased from Thermo Fisher Scientific (Waltham, MA, USA). The enzyme-linked immunosorbent assay (ELISA) sets for inflammatory-related cytokine detections in both animal models and in vitro tests were purchased from BD Biosciences (San Diego, CA). All of the reagents for cell culture were from Hyclone (Logan, UT, USA).

### LL-37-induced rosacea-like animal model

The mouse model of rosacea was adopted from Yamasaki et al. (2007). The experimental protocol was approved by the Kaohsiung Medical University Institutional Animal Care and Use Committee (IACUC number 106015). Briefly, 4-5 weeks-old male BALB/c mice (National Laboratory Animal Center, Taipei, Taiwan) were weighed and grouped to minimize group weight differences for naïve control (NA), phosphate-buffered saline (PBS)-treated group (LL-37), and CB1-treated group (LL-37 + CB1) after one-week acclimatization. Each animal was marked 4-6 positions on the dorsal area. Except for the NA group, mice were injected with 40 µL of LL-37 (320 µM) intradermally twice daily for 2 days at the marked positions on the back. The CB1 (4 mg/mL) or PBS were intraperitoneally administered (100 µL/20 g) to mice daily for two days after the first and the third inductions. For each mouse, the marked positions were photographed and harvested a day after the last LL-37 induction. The skin sections were processed for the histological examination, myeloperoxidase (MPO) measurements, MIP-2 measurements, or mRNA extraction. The mice were anesthetized by isoflurane (2%) before harvesting the skin sections and euthanization.

### Histological examination of the skin section

The marked mice skin sections were harvested and fixed with 4% neutral formalin buffer for at least 1 day. The skin sections were embedded in paraffin and cut into 3 µm thick slices. The sections were stained with hematoxylin and eosin or processed for immunohistochemistry staining for Gr-1, rat anti-mouse polyclonal antibody (R&D Systems, Minneapolis, MN, USA). Each skin section was separated into 4-6 fields at 200-fold magnification; the numbers of neutrophils were counted manually from each field then normalized by the field number.

### RNA isolation and real-time reverse transcription-polymerase chain reactions (RT-PCR)

Total RNA was isolated from the skin samples by isol-RNA lysis reagent (5-Prime, San Francisco, CA, USA). The total RNA of each sample was reverse-transcribed to cDNA by RevertAid RT Kit (Thermo Fisher Scientific, Waltham, MA, USA) using oligo (dT). The expression of the genes was quantified using SYBR Mix (Thermo Fisher Scientific, Waltham, MA, USA) by Real-time PCR 7900 (Applied Biosystems, Foster City, CA, USA). The assay reactions were 95°C for 10 min, followed by 40 cycles of 95°C for 15 s and 60°C for 1 min. The fold-changes of the genes between groups were calculated by the ΔΔCt method. The primers used in this study included HPRT (5′-TCAGTCAACGGGGGACATAAA-3′ and 5′-GGGGCTGTACTGCTTAACCAG-3′) (Shi et al., 2010), MIP-2 (5′-AGTGAACTGCGCTGTCAATGC-3′ and 5′-GCCCTTGAGAGTGGCTATGACT-3′) (Mukhopadhyay et al., 2011), and KC (5′-CAGCCACCCGCTCGCTTCTC-3′ and 5′-TCAAGGCAAGCCTCGCGACCAT-3′) (Degos et al., 2015)**.**

### Cytokine measurements

The skin samples were harvested and homogenized with 5-fold tissue weight volume of lysis buffer (10 mM pH 8.0 Tris–HCl buffer with 150 mM NaCl, 1% Tween 20, 10% Glycerol, 5 mM EDTA) and 200:1 diluted protease inhibitor cocktail solution (Merck Millipore, Danvers, MA, USA). After centrifugation, the supernatants were quantified for macrophage inflammatory protein 2 (MIP-2) by ELISA kit (R&D Systems, Minneapolis, MN, USA).

### Myeloperoxidase (MPO) activity assay

The MPO activity, as an indicator of the total inflammation level of the lesions, was adopted from protocols published previously (Queiroz et al., 2009). Briefly, the skin sections were homogenized in one mL cooled phosphate buffer (pH 4.7) supplemented with a 5 µL Protease inhibitor cocktail set solution (Merck Millipore, Danvers, MA, USA). After centrifuging the homogenized sample, the sample buffer was replaced by 0.05 M sodium phosphate buffer (pH 5.4 , containing 0.5% hexadecyltrimethylammonium bromide (HTAB) solution). After three times frozen-thawed cycles (frozen at −80°C for 8 min and thawed at room temperature for 10 min), the samples were centrifuged and the supernatants were collected for MPO evaluation. For MPO quantification, 12.5 µL of the samples and diluted standards were mixed with 62.5 µL of 3,3′, 5,5′-tetramethylbenzidine (TMB) solution (1.6 mM TMB, 0.0045% H_2_O_2_, 0.5% HTAB solution) in 96-well plates. The plates were then incubated at room temperature for 5–45 min, and the reaction was stopped by adding 50 µL of the stop solution (4 M H_2_SO_4_). The OD values at 450 nm/570 nm were recorded. The amount of myeloperoxidase was normalized by the amount of total protein in each sample.

### Cell culture

Human keratinocyte HaCaT was from Prof. Yen, Kaohsiung Medical University (Huang et al., 2019), and monocyte THP-1 cell line was obtained from Bioresource Collection and Research Center (BCRC). HaCaT and THP-1 cells were maintained in Dulbecco’s Modified Eagle Medium (DMEM) and RPMI 1640, respectively, supplemented with 10% fetal bovine serum (FBS) and 1% penicillin/streptomycin. Cells were seeded to antibiotics free and low/no FBS (0% in HaCaT cells, and 2% in THP-1 cells) culture medium for the experiments.

### In vitro cell experiments

HaCaT cells (7 × 10^4^ cells/mL, 300 µL/well) and THP-1 cells (1 × 10^6^ cells/mL, 300 µL/well) were either left untreated (control) or stimulated with LL-37 (5 µM) in the absence or the presence of CB1 (1, 10, and 20 µM) for 24 h in 48-well plates. After 24 h co-incubation, supernatants were collected for cytokine/chemokine measurements by ELISA (R&D Systems, Minneapolis, MN, USA) as recommended by the manufacturer. The cells were harvested for cell viability by 3-(4,5-dimethylthiazol-2-yl)-2,5-diphenyltetrazolium bromide (MTT) assay following the protocol from a previous study (Cheng et al., 2017).

For Western blotting, HaCaT cell (1×10^5^ cells/mL, 10 mL/10cm dish) and THP-1 cell (1×10^6^ cells/mL, 10 mL/10cm dish) were either untreated (NA) or stimulated with 5 µM LL-37 and CB1 (0, 1, 10 µM). Briefly, the cells were lysed by radioimmunoprecipitation assay buffer (RIPA, 20 mM Tris–HCl, 150 mM NaCl, 1 mM EDTA, 1 mM ethylene glycol tetra-acetic acid, 1% Triton X-100, and 1 mM phenylmethylsulfonyl fluoride, pH 7.4) and heated with 4X sodium dodecyl sulfate (SDS) sample buffer (8% SDS, 0.2 M Tris (pH 6.8), 0.4 M dithiothreitol, 0.4% coomassie brilliant blue R-250, and 40% glycerol) at 95°C for 5 min. The lysates were resolved by 10% SDS-PAGE and transferred to polyvinylidene fluoride membrane (Millipore, Bedford, MA, USA, 0.45 µM). The membranes were blocked with 5% skim milk in Tris buffer saline containing 0.1% Tween-20 (TBST) under 60 rpm rocking at room temperature for 1 h. Membranes were blotted with the primary antibodies in sequence: Phosphorylation protein (Phospho-ERK or Phospho-p38), total protein (total-ERK or total-p38), and internal control protein (GAPDH). The primary antibodies were diluted in TBST supplemented with 5% bovine serum albumin (BSA) or 5% skim milk. The membranes were washed three times with TBST under 120 rpm rocking at room temperature after overnight incubation with a primary antibody under 60 rpm rocking at 4°C. After blotting with the secondary antibody conjugated with horseradish peroxidase (HRP) in TBST with 5% skim milk for 1 h at room temperature, the activity of HRP was developed and detected using enhanced chemiluminescence (Thermo Fisher Scientific, Waltham, MA, USA). Images were captured by using Chemi-Doc Gel Imaging System (Bio-Rad Laboratories, Inc., Hercules, CA, USA). The antibodies were all purchased from Cell Signaling Technology (Danvers, MA, USA). The phospho-antibodies phospho-ERK and phospho-p38 recognize residues surrounding Thr202/Tyr204 of human p44 MAPK and Thr180/Tyr182 of human p38 MAPK, respectively.

### Statistical analysis

The statistical differences between the CB1 treatment group and the LL-37-only group were analyzed by parametric analysis of variance (ANOVA) with Dunnett’s test by Prism 5.0 (GraphPad Software Inc., USA). *P* <0.05 was defined as statistical significance.

## Results

### CB1 attenuated LL-37-induced skin redness and local inflammation responses in vivo

**Figure 1:**
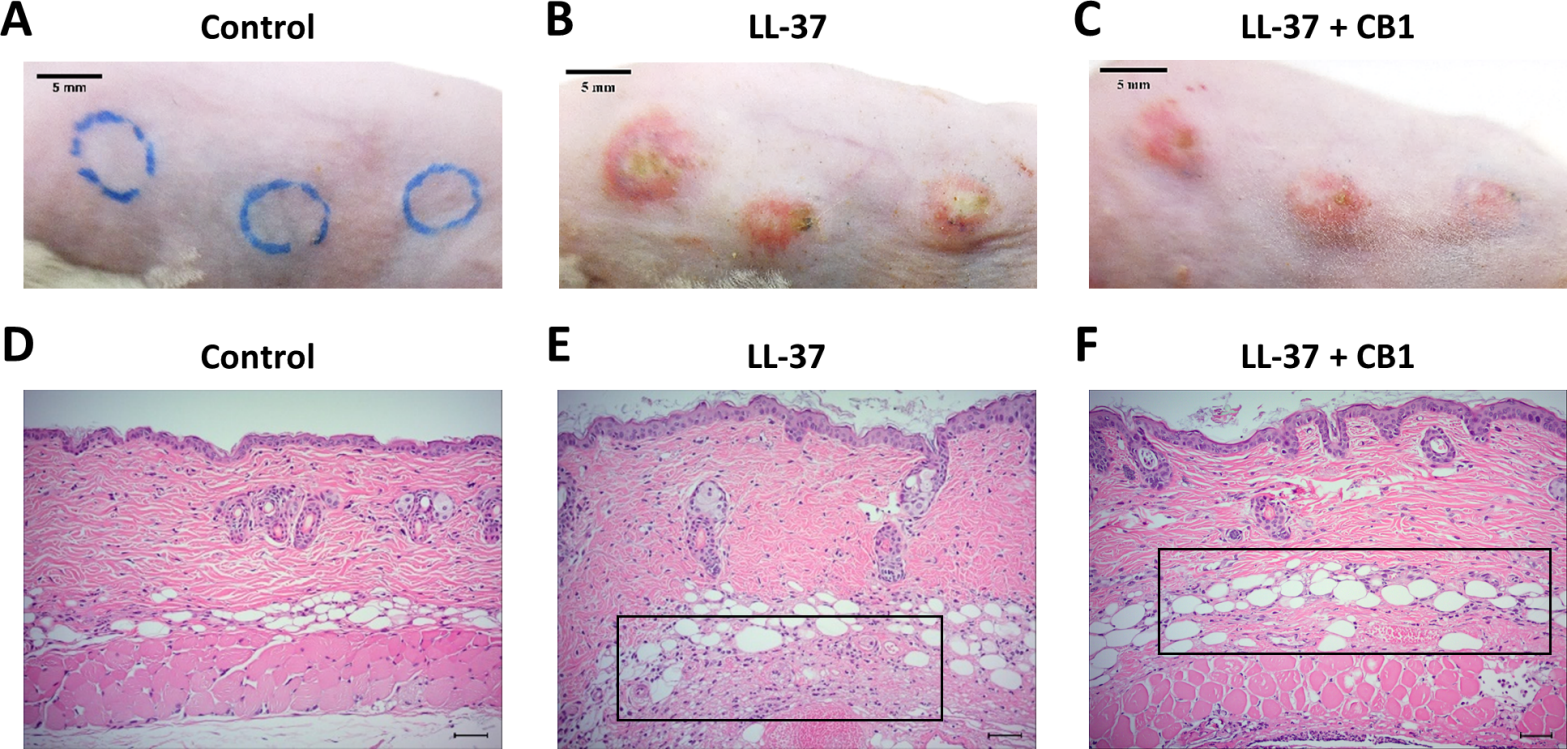
CB1 attenuated the signs of the rosacea-like lesions in the LL-37-induced rosacea-like mouse model. (A–C) skin signs; (D–F) immune cell recruitment.The rosacea-like lesions were induced by intradermal LL-37 (320 µM) injections twice daily for 2 days. Cinnamtannin B1 (20 mg/kg) or its solvent, PBS, were intraperitoneally administered to mice daily for two days. The photos and skin sections were taken one day after the last LL-37 induction from three independent experiments. The inset boxes indicate the main area of the cell necrosis (incomplete cells) and the immune cell infiltration (bright purple signals).

**Figure 2:**
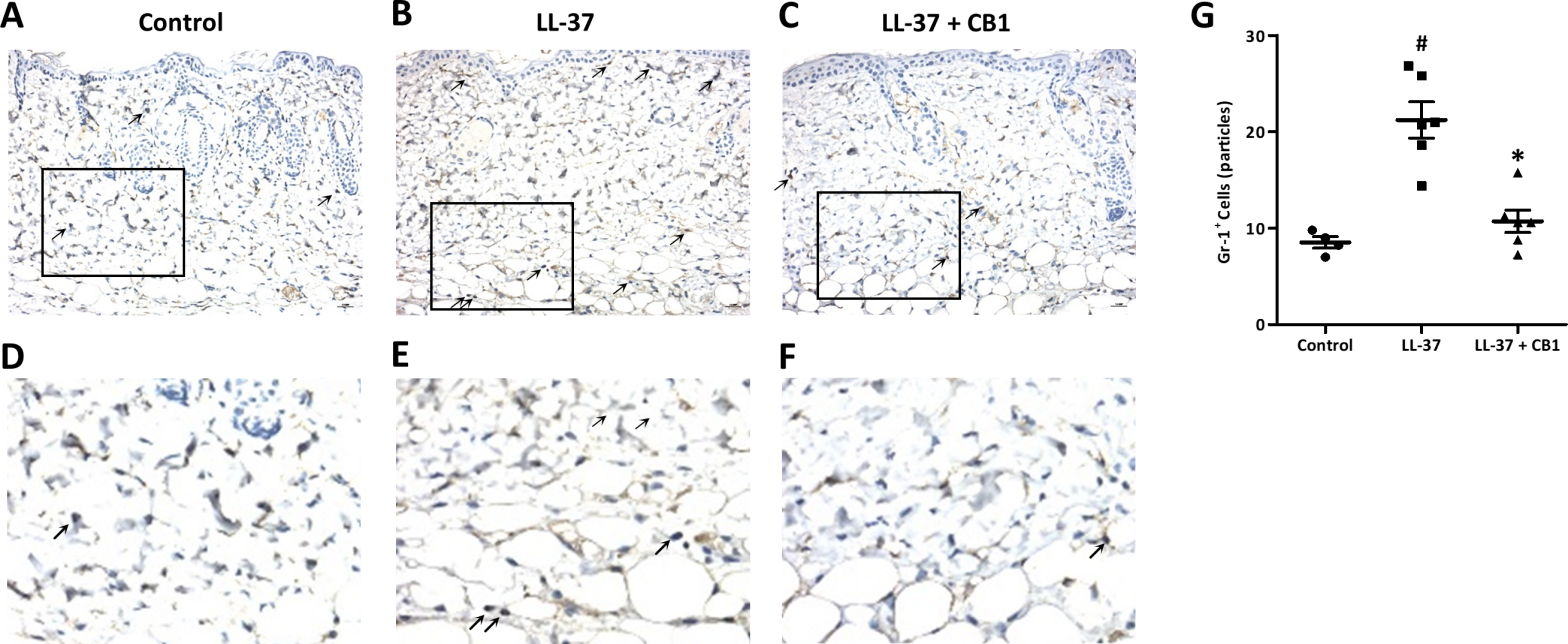
CB1 reduced neutrophils recruitment to the rosacea-like skin lesions. (A–F) Immunohistochemistry with Gr-1^+^ antibody and (G) quantitative results of the Gr-1^+^-cells in the sections. (A–F) Staining of the normal control skin (Control), LL-37-injected skin (LL-37) and LL-37-injected skin with CB1 for treatment (LL-37 + CB1). The skin sections were stained with anti-mouse Gr-1 antibody for examining polymorphonuclear neutrophils as described in the methods. The micrographs were shown at 200X magnification, and the arrows indicated the Gr-1 positive cells. (G) The number of Gr-1^+^ cells was counted manually. Scale bars represented 50 µm at histology. Data are expressed as the mean ±standard error of the mean from three independent experiments (Control *n* = 4, LL-37 *n* = 6, LL-37 + CB1 *n* = 6). ^#^*p* < 0.05, the LL-37-induced group compared to the control group. ^∗^*p* < 0.05, the CB1 treatment group compared to the LL-37-induced group.

LL-37 induced rosacea-like signs, characterized by cutaneous erythema with central necrosis, at the sites of injection, while the CB1 treatment reduced the LL-37-induced rosacea-like signs (Figs. 1A–1C). Histological sections of the lesions showed that LL-37 induced neutrophil infiltration, hemorrhage, thickening and cell necrosis in the epidermal tissues, and these observations appeared to be reduced in the CB1 group (Figs. 1D–1F). Immunohistochemistry staining of the skin sections with Gr-1 for polymorphonuclear neutrophils indicated that LL-37 induced neutrophil recruitment to the skin, and the CB1 treatment decreased the neutrophil recruitment (Figs. 2A–2F). The number of GR-1^+^ cells was significantly higher than the controls after LL-37 induction and the number was decreased by CB1 (Fig. 2G).

### CB1 suppressed MPO and MIP-2 production in vivo

**Figure 3:**
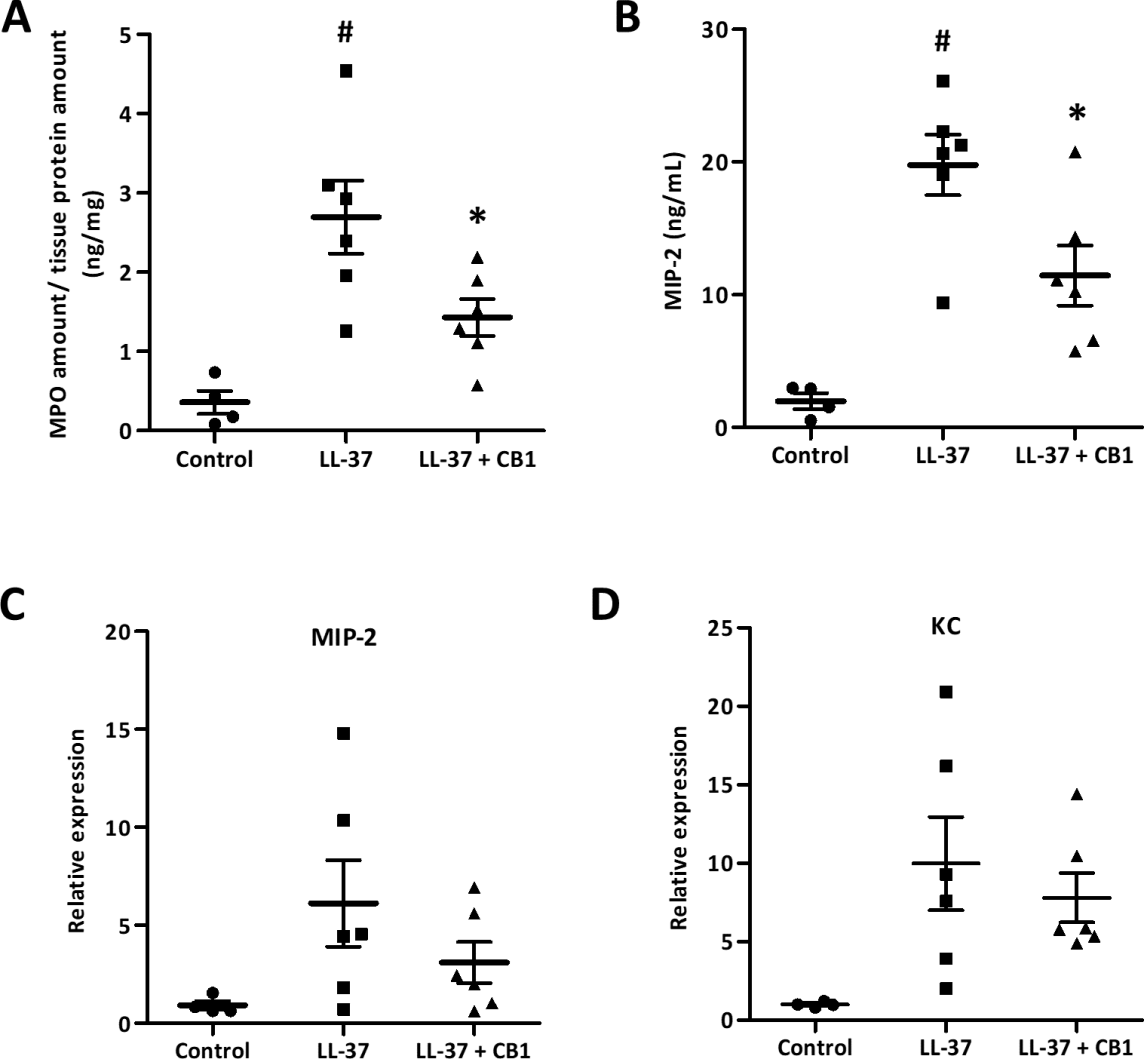
CB1 attenuated LL-37-induced inflammatory and neutrophil chemoattractant MIP-2 at both protein and gene levels in the rosacea-like mouse model. (A) Myeloperoxidase (MPO) (B) MIP-2 protein (C) MIP-2 mRNA expression (D) KC mRNA expression. The protein levels of MPO and MIP-2 were measured by MPO activity assay and ELISA, respectively. The gene expression levels were detected by qPCR analysis. Data are expressed as the mean ± standard error of the mean from three independent experiments (Control *n* = 4, LL-37 *n* = 6, LL-37 + CB1 *n* = 6). ^#^*p* < 0.05, the LL-37-induced group compared to the control group. ^∗^*p* < 0.05, the CB1 treatment group compared to the LL-37-induced group.

The skin sections were homogenized for further examination of inflammatory responses. MPO, a biomarker for overall inflammation, was elevated in the LL-37-induced lesion, and the CB1 treatment reduced the LL-37-induced MPO level (Fig. 3A). MIP-2 and KC are functional homologs of neutrophil chemoattractant IL-8 in mice. The elevated levels of MIP-2 in the LL-37-induced lesions were also attenuated by CB1 treatment (Fig. 3B). MIP-2 and KC gene expression profiles although were not significantly decreased by CB1, they showed similar trends as the MIP-2 levels (Figs. 3C & 3D).

### CB1 reversed LL-37-induced IL-8 production by HaCaT and THP-1 cells in vitro

Since the recruitment of neutrophils and production of MIP-2 (mouse functional homologs of IL-8) were both significantly attenuated by administration of CB1 in vivo, the effects of CB1 on human cells were further studied in human keratinocyte HaCaT and human monocyte THP-1 cells in vitro.

**Figure 4:**
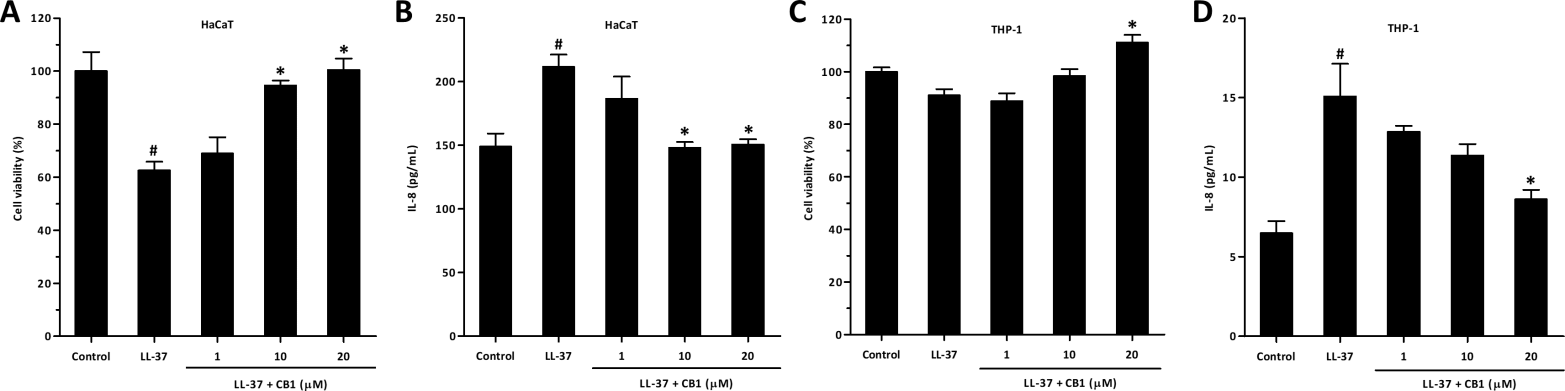
CB1 reversed LL-37-induced interleukin-8 (IL-8) production by HaCaT and THP-1 cells in vitro. (A) HaCaT cell viability (B) HaCaT IL-8 production (C) THP-1 cell viability (D) THP-1 IL-8 production. HaCaT cells and THP-1 cells were either left untreated (control) or stimulated with LL-37 (5 µM) in the absence or the presence of CB1 (1, 10, 20 µM) for 24 h. The experiments were performed at least 3 times in independent experiments. The bar graph presented results from a representative experiment (*n* = 4). Data are expressed as mean ± standard error of the mean. ^#^*p* < 0.05, LL-37-stimulated cells compared to the cell alone (control group). ^∗^*p* < 0.05, different concentrations of CB1 in the presence of LL-37 compared to the LL-37-stimulated cells.

LL-37 significantly decreased the viability of HaCaT cells at 5 µM (Fig. 4A), and CB1 at the concentration of 10 µM reversed the LL-37-induced cytotoxicity. By contrast, LL-37 did not affect the viability of THP-1 at 5 µM (Fig. 4C). LL-37 significantly induced IL-8 production both in HaCaT and THP-1 cells, and CB1 reversed the LL-37-induced IL-8 secretion (Fig. 4B and Fig. 4D).

### CB1 inhibited LL-37-induced IL-8 production through extracellular signal-regulated kinase (ERK) mitogen-activated protein kinase (MAPK) pathway

**Figure 5:**
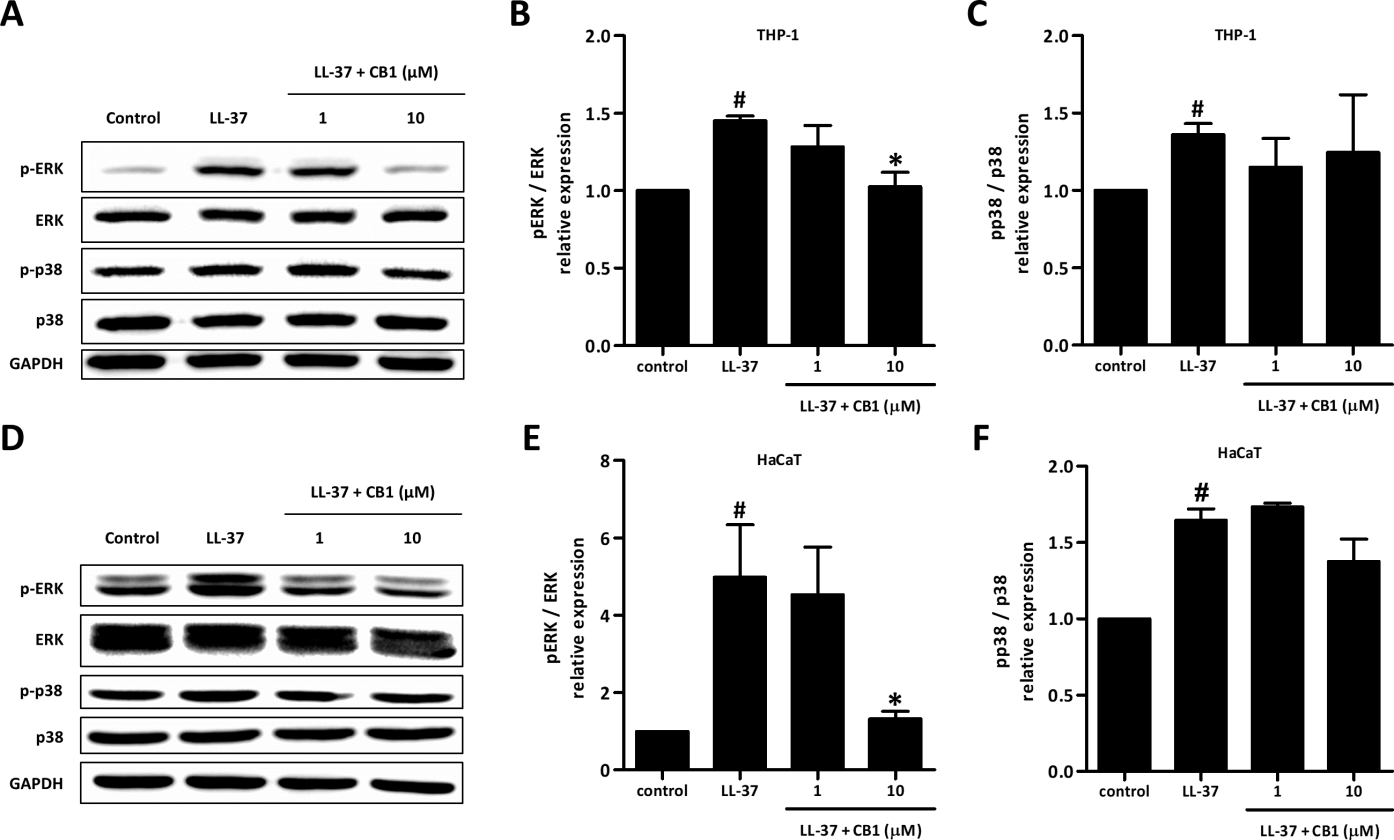
CB1 down-regulated the phosphorylation of ERK but not p38 of the mitogen-activated protein kinase (MAPK) pathway induced by LL-37 in vitro in THP-1 cells (A–C) and HaCaT cells (D–F). HaCaT cells and THP-1 cells were pretreated with CB1 (1 and 10 µM) for 30 min followed by LL-37 (5 µM) stimulation for 15 min. The Western blotting showed the results from a representative experiment. The bar figures presented the mean and standard error of the mean from three independent experiments. ^#^*p* < 0.05, LL-37-induced cells compared to the control group. ^∗^*p* < 0.05, CB1 and LL-37 treated cells compared to the LL-37-induced cells only.

LL-37 up-regulated the phosphorylation of ERK and p38 both in HaCaT and THP-1 cells. CB1 at 10 µM reversed the LL-37-induced phosphorylation of ERK but not p38 (Figs. 5B and 5E).

## Discussion

In this study, we demonstrated CB1 can reverse LL-37-induced pro-inflammatory responses both in vitro and in vivo. CB1 was able to attenuate the severity and the inflammation of rosacea-like lesions in the mouse model. CB1 decreased the expression of MIP-2, the cytokine functionally similar to human IL-8, and may therefore result in fewer neutrophils at the lesions. CB1 was also shown to decrease the production of IL-8 induced by LL-37 in human keratinocytes and monocytes in vitro. The anti-inflammatory effect of CB1 was in part due to the decreased phosphorylation of ERK, but not p38, in the MAPK pathway.

Rosacea is a multifaceted inflammatory disease. The pathophysiology of rosacea is not fully understood. Various inflammatory factors, factors causing vascular changes, and certain neuropeptides which can mediate dysregulation of inflammation are also found enhanced locally. Infiltration of a mixed population of macrophages, mast cells, and neutrophils has commonly been described in biopsies taken from rosacea patients (Gerber et al., 2011; Buhl et al., 2015). In the papulopustular stage of rosacea, neutrophils often become dominant (Gerber et al., 2011; Buhl et al., 2015). LL-37 was one of the factors shown to play important roles in the inflammation in rosacea (Yamasaki et al., 2011; Chen et al., 2017). Yamasaki and colleagues (Yamasaki et al., 2007) reported that the cathelicidin levels in lesional skin of rosacea patients were significantly higher than normal skin (∼1100 [800-1500] µM vs. 100 µM). The intradermal injections of LL-37 at 320 µM, although lower than the LL-37 levels observed in human rosacea lesions, induced rosacea-like inflammatory reactions in the mouse model. The model has been successfully utilized to show the involvement of mast cells in the LL-37-induced inflammation (Kim et al., 2017; Chen et al., 2017). In this study, CB1 attenuated the severity of redness, inflammation, and neutrophil infiltration at the lesions in the LL-37-induced rosacea-like inflammation in the mice, indicating the effects of CB1 on LL-37-induced inflammation. Topical administration would be expected to achieve higher concentration at the epidermis and cause less systemic adverse effects. Given the effectiveness of CB1 in the mice model, future development in topical formulations of CB1 may be warranted.

LL-37 at 5–10 µM was shown to induce IL-8 production in keratinocyte cells (Boink et al., 2017), neutrophils and monocytes (Yang et al., 2000; Yang et al., 2020a; Yamasaki et al., 2007; Bucki et al., 2010). In addition, LL-37 was shown to be a direct leukocyte chemoattractant for neutrophils and monocytes (Hemshekhar, Choi & Mookherjee, 2018). We hypothesized that elevated levels of LL-37 may exaggerate the inflammatory in rosacea through inducing inflammation and attracting neutrophils and other immune cells to the lesions. The in vitro data in our study revealed that CB1 can directly decrease LL-37-induced IL-8 production by keratinocytes. This attenuation of local inflammation by CB1 may be one of the mechanisms by which CB1 attenuated the LL-37-induced rosacea-like signs.

LL-37 at 10 µM is known to activate neutrophils, monocytes, and epithelial cells similarly via interacting with the formyl peptide receptor-like 1 (FPRL1) receptor (Yang et al., 2000). LL-37 has been known to induce inflammatory responses in epithelial cells and monocytes by activation of mitogen-activated protein kinase (MAPK) pathway through phosphorylation of ERK1/2 and p38 kinases but not JNK (Niyonsaba et al., 2005). Inhibition of either one of the kinases could reduce LL-37-induced IL-8 production and downregulate the transcription of various chemokine genes (Bowdish et al., 2004). In addition, LL-37 has been suggested to suppress neutrophil apoptosis through ERK1/2 phosphorylation (Nagaoka, Tamura & Hirata, 2006). This inhibition of neutrophil apoptosis may worsen tissue inflammation and damage. Our data showed that CB1 significantly suppressed LL-37-induced phosphorylation of ERK but not p38 both in keratinocytes and monocytes indicating the differential effects of CB1 on LL-37-induced activation of downstream signaling pathways. Therefore, CB1 may also limit the inflammation by reversing the LL-37-induced neutrophil apoptosis inhibition.

Several genes downstream of MAPK signaling pathways, including AP1, MYC-MAX, NFKB1, SP1, ElK1, were identified to be activated by LL-37 stimulation in monocytes (Bowdish et al., 2004; Mookherjee et al., 2009). An active form of nuclear factor kappa B (NF-κB) was found to be elevated in rosacea patients, implying the involvement of NF-κB in the pathogenesis of rosacea (Wladis, Lau & Adam, 2019). The activation of AP-1 and MYC-MAX has also been indicated to play roles in epithelial proliferation and differentiation, which may result in impairment of the stratum corneum barrier function (Gebhardt et al., 2006; Addor, 2016). These genes may be used to further elucidate the therapeutic effects of CB1 and for the development of other therapy for rosacea.

Due to the multifactorial nature of rosacea, the mechanisms of action of many effective rosacea therapies are not fully clear. Inflammation appears to play a central role in rosacea. Many agents proven to be effective in rosacea symptoms, for instance azelaic acid, retinoids, metronidazole, tetracyclines, and ivermectin, all have certain levels of anti-inflammatory activity (Pelle, Crawford & James, 2004; Jones, 2009; Fallen & Gooderham, 2012; Cardwell et al., 2016). A few phytochemicals with anti-inflammatory activity such as licochalcone A, silymarin, *Chrysanthellum indicum* extract, and *Quassia* wood extract have also been shown to relieve rosacea symptoms (Rigopoulos et al., 2005; Sulzberger et al., 2016; Saric et al., 2017).

CB1 as well as botulinum toxin and artemisinin were shown to reduce LL-37-induced inflammation (Choi et al., 2019; Yuan et al., 2019) in the animal model but not yet been evaluated in humans. Although the LL-37-induced inflammation may not represent all factors causing rosacea, the association between LL-37 and rosacea-like symptoms makes the effectiveness of these agents in patients with rosacea worth further evaluation. The development of alternative therapeutic agents with diverse mechanisms for reducing inflammation in rosacea could benefit patients not suitable for certain treatments or not tolerant to certain adverse effects. The development of newer agents for these skin diseases could also reduce the use of antibiotics for these skin diseases and thereby reduce drug-resistant problems associated with overuse of antibiotics for their anti-inflammatory effects.

Furthermore, many of the agents used to manage rosacea symptoms also show effects in acne. This may be due to the shared characteristics in inflammation by these two skin diseases. Given CB1 also has some anti-bacterial activities against *P. acnes* in addition to its anti-inflammatory activity, CB1 may also be effective in acne. Our preliminary data also support this theory (Yang et al., 2020b). In addition, LL-37 was also suggested to play roles in psoriasis, atopic dermatitis and nickel allergy (Chen et al., 2006). Ong et al. (2002) reported that the level of LL-37 concentration in the psoriatic lesions ranged widely from 0 to 1605 µM (median 304 µM), and the mRNA levels of LL-37 in atopic lesions were significantly lower than psoriatic lesions. Although it is not clear whether the elevated levels of LL-37 were the causation or the result of these skin conditions, these observations indicated that LL-37 may play some roles in lesion inflammation in all these diseases. Therefore, CB1 may also have potential as a therapy for these diseases. Further evaluations should be warranted.

## Conclusion

CB1 showed potential as a therapy for rosacea. CB1 attenuated LL-37 induced redness, local inflammation, and neutrophil infiltration in the LL-37-induced rosacea-like mouse model. CB1 appeared to achieve the effect by inhibiting IL-8 production and the phosphorylation of the ERK/MAPK pathway in human cell lines of keratinocytes and monocytes.

##  Supplemental Information

10.7717/peerj.10548/supp-1 Supplemental Information 1 Chemical structure of Cinnamtannin B1.

10.7717/peerj.10548/supp-2 Supplemental Information 2 Raw data, including all in vivo. tests and in vitro tests (Figs. 2–4). These data were used for statistical analysis

10.7717/peerj.10548/supp-3 Supplemental Information 3 The full-length uncropped blots from Fig. 5

## Abbreviations

ANOVA: analysis of variance
BCRC: Bioresource Collection and Research Center
BSA: bovine serum albumin
CB1: cinnamtannin B1
COX-2: cyclooxygenase-2
DMEM: Dulbecco’s Modified Eagle Medium
ELISA: enzyme-linked immunosorbent assay
ERK: extracellular signal-regulated kinase
FBS: fetal bovine serum
FPRL1: formyl peptide receptor-like 1
HRP: horseradish peroxidase
HTAB: hexadecyltrimethylammonium bromide
IACUC: Institutional Animal Care and Use Committee
IL-8: interleukin-8
MAPK: mitogen-activated protein kinase
MIP-2: macrophage inflammatory protein 2
MPO: myeloperoxidase
NA: naïve
PBS: phosphate-buffered saline
PT-PCR: reverse transcription-polymerase chain reactions
RIPA: radioimmunoprecipitation assay buffer
ROS: reactive oxygen species
SDS: sodium dodecyl sulfate
TBST: tris buffer saline containing 0.1% Tween-20

## Additional Information and Declarations

### Data Availability

The following information was supplied regarding data availability: Raw data, including all in vivo tests and in vitro tests, are available in the Supplemental Files.
