# Supplementary figures and images for: Cinnamtannin B1 attenuates rosacea-like signs via inhibition of pro-inflammatory cytokine production and down-regulation of the MAPK pathway
Source: PeerJ. 2020 Dec 21;8:e10548. doi: 10.7717/peerj.10548 (PMC7759128; doi:10.7717/peerj.10548)

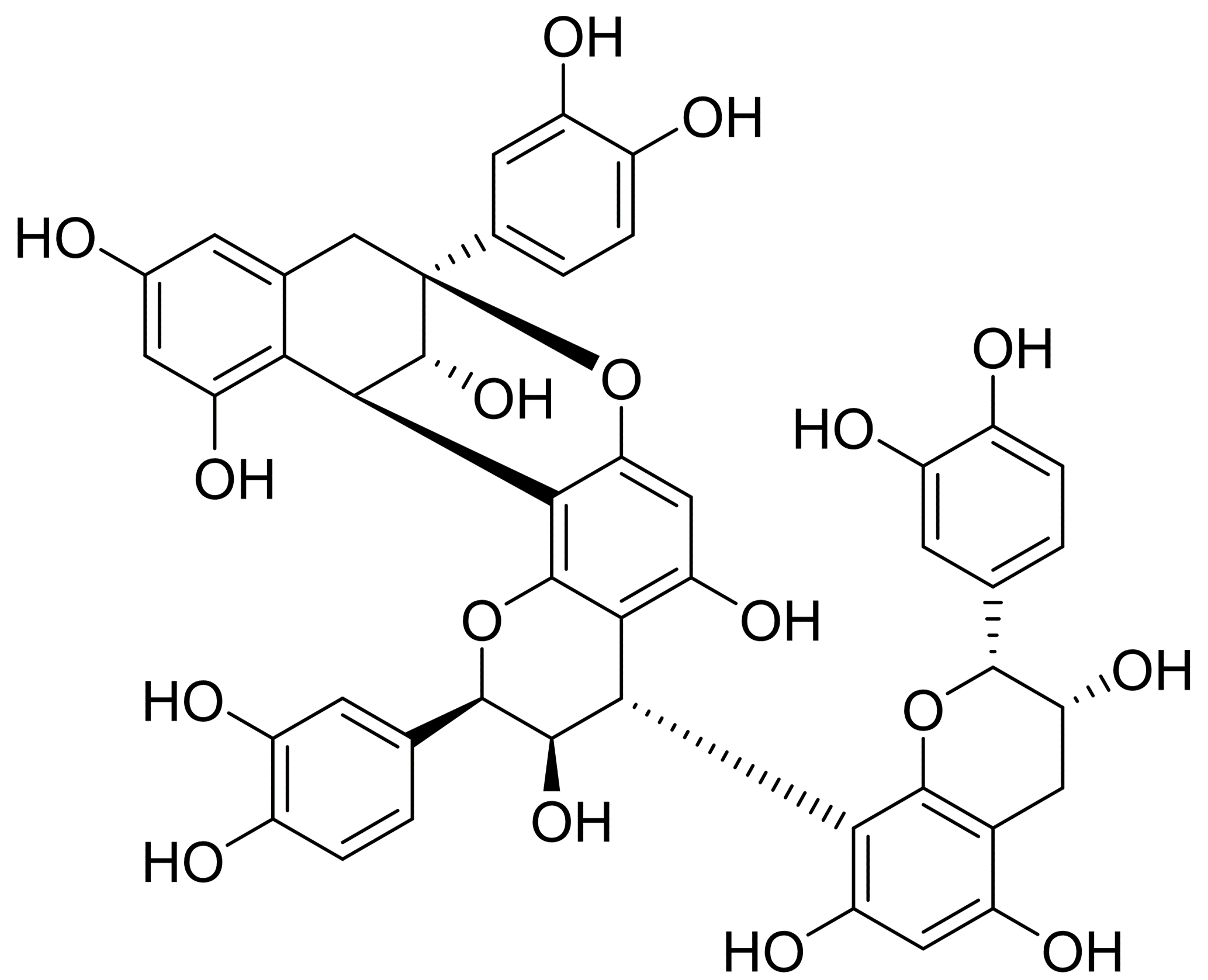

Supplement: Supplemental Information 1 [file peerj-08-10548-s001.png]

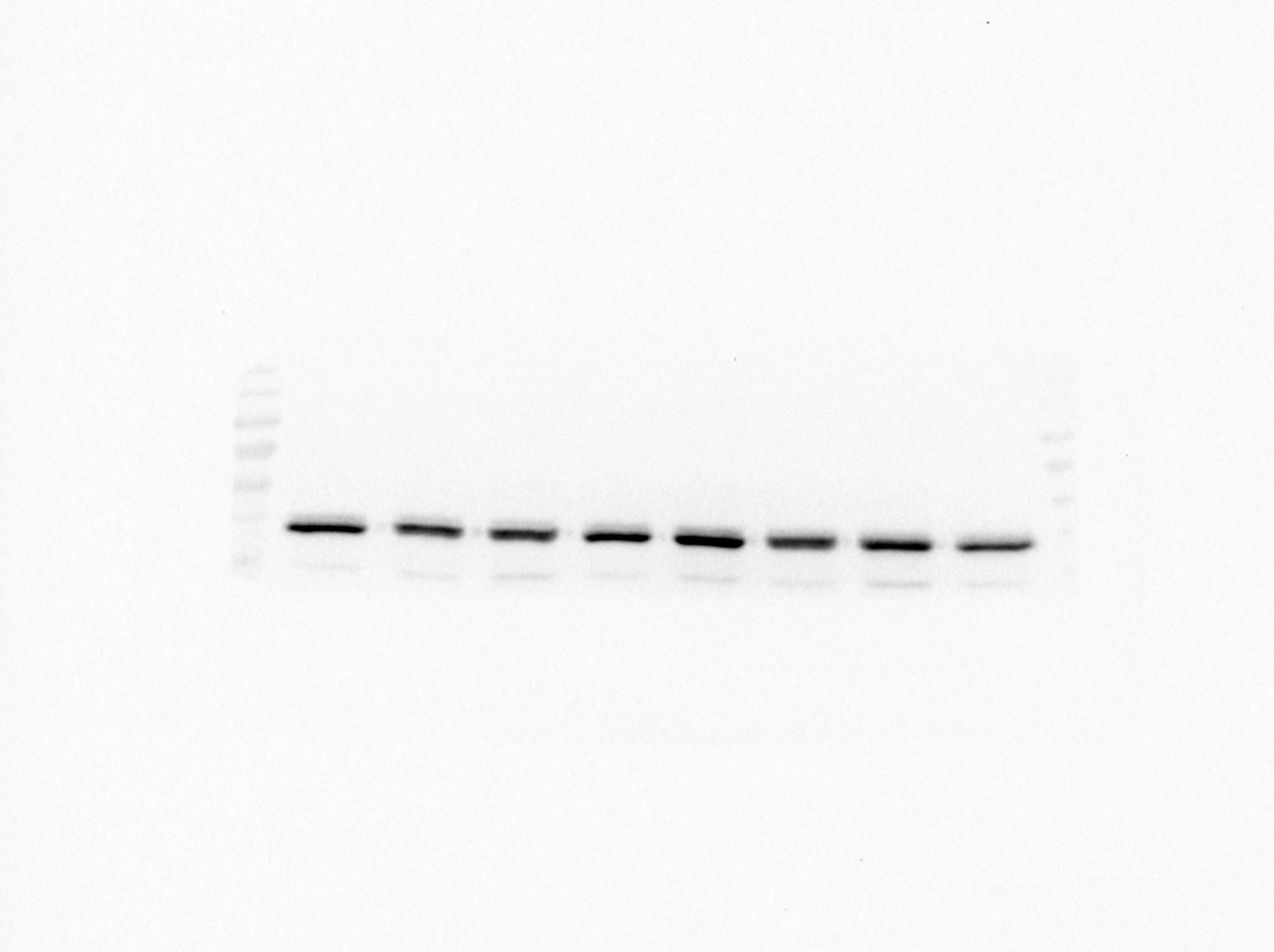

Supplement: Supplemental Information 3 [file peerj-08-10548-s003.zip › Supplemental files (Figure 5)/HaCaT/HaCaT-ERK.jpg]

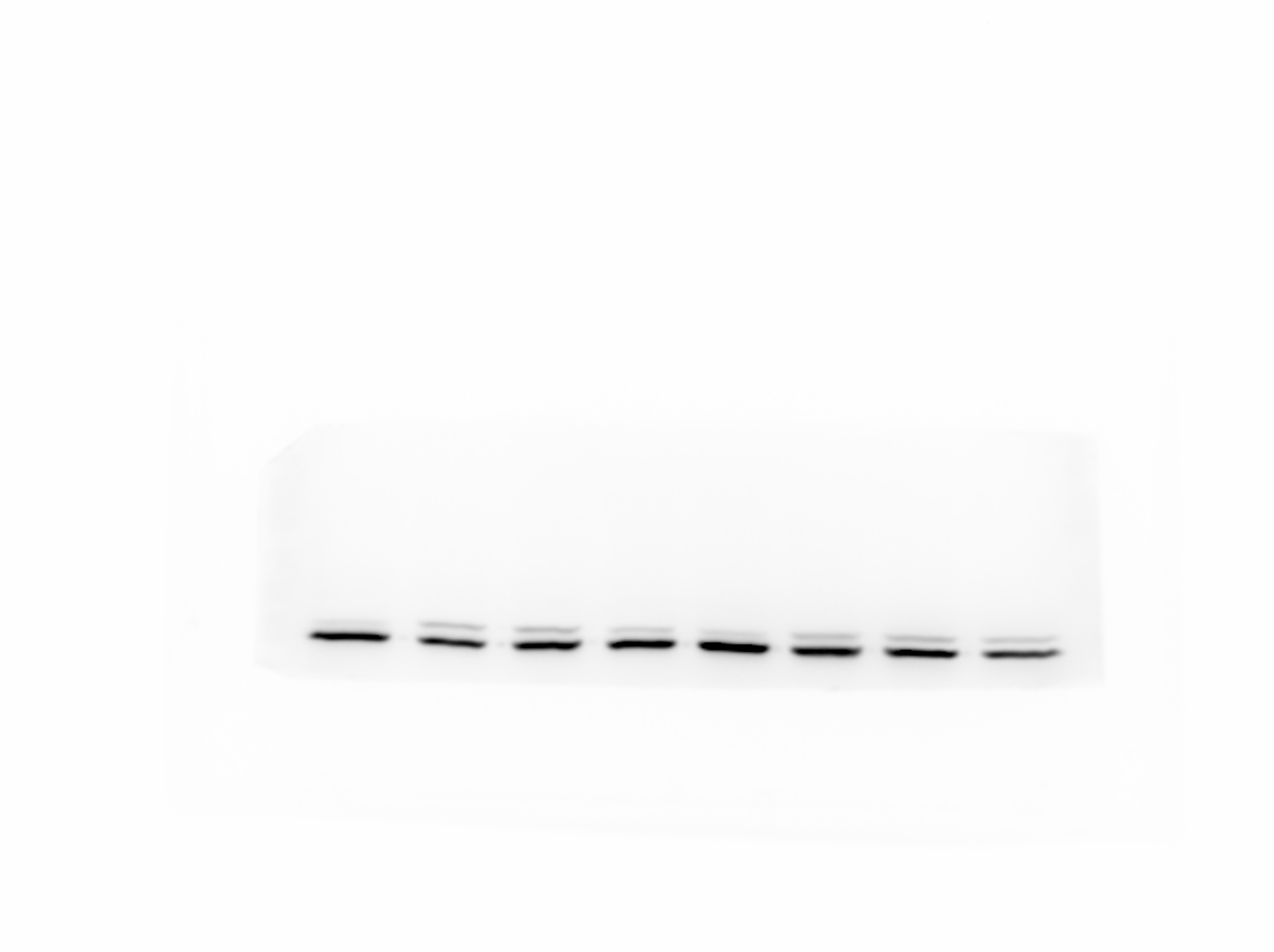

Supplement: Supplemental Information 3 [file peerj-08-10548-s003.zip › Supplemental files (Figure 5)/HaCaT/HaCaT-GAPDH (ERK).jpg]

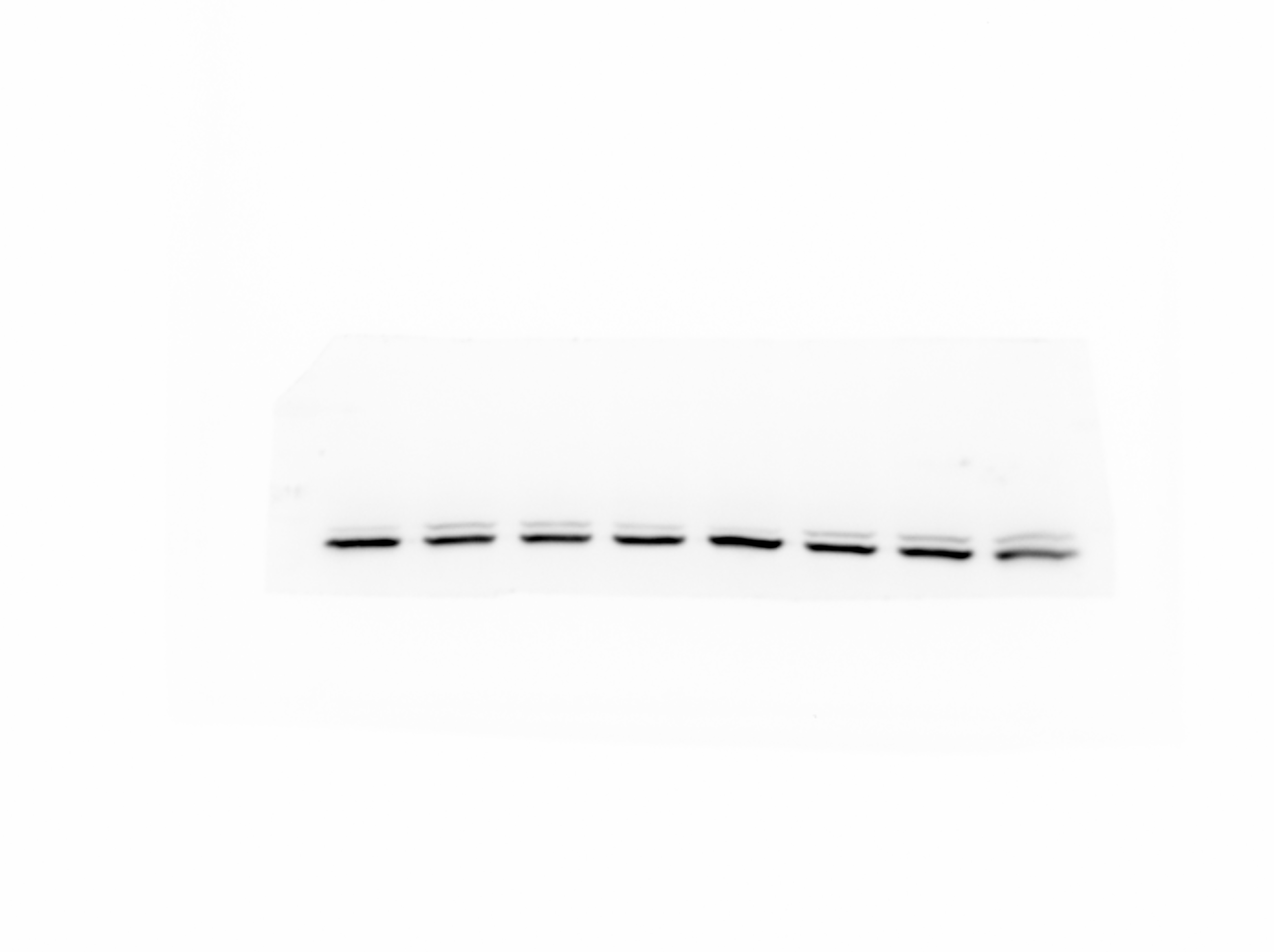

Supplement: Supplemental Information 3 [file peerj-08-10548-s003.zip › Supplemental files (Figure 5)/HaCaT/HaCaT-GAPDH (p38).jpg]

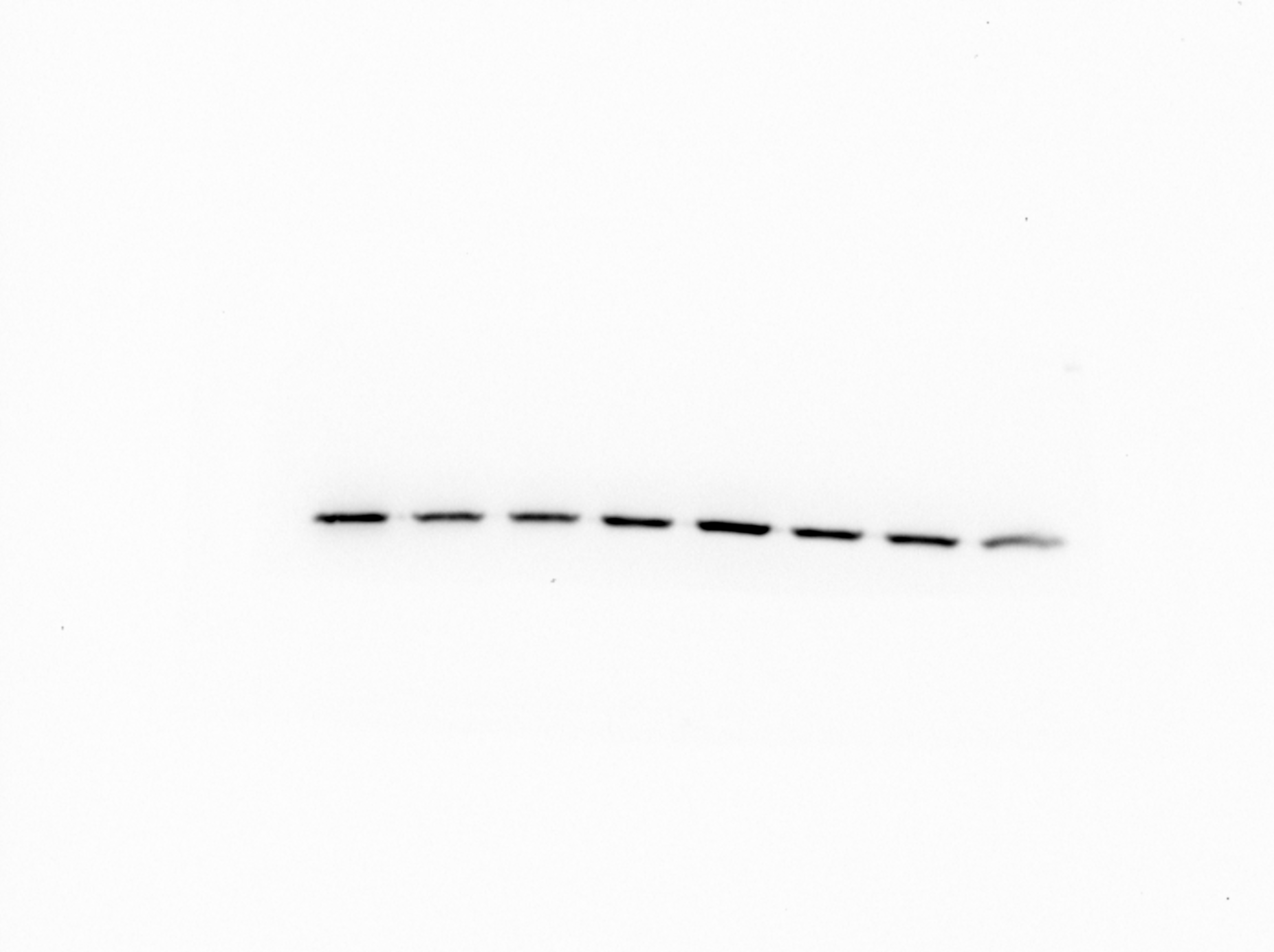

Supplement: Supplemental Information 3 [file peerj-08-10548-s003.zip › Supplemental files (Figure 5)/HaCaT/HaCaT-p38.jpg]

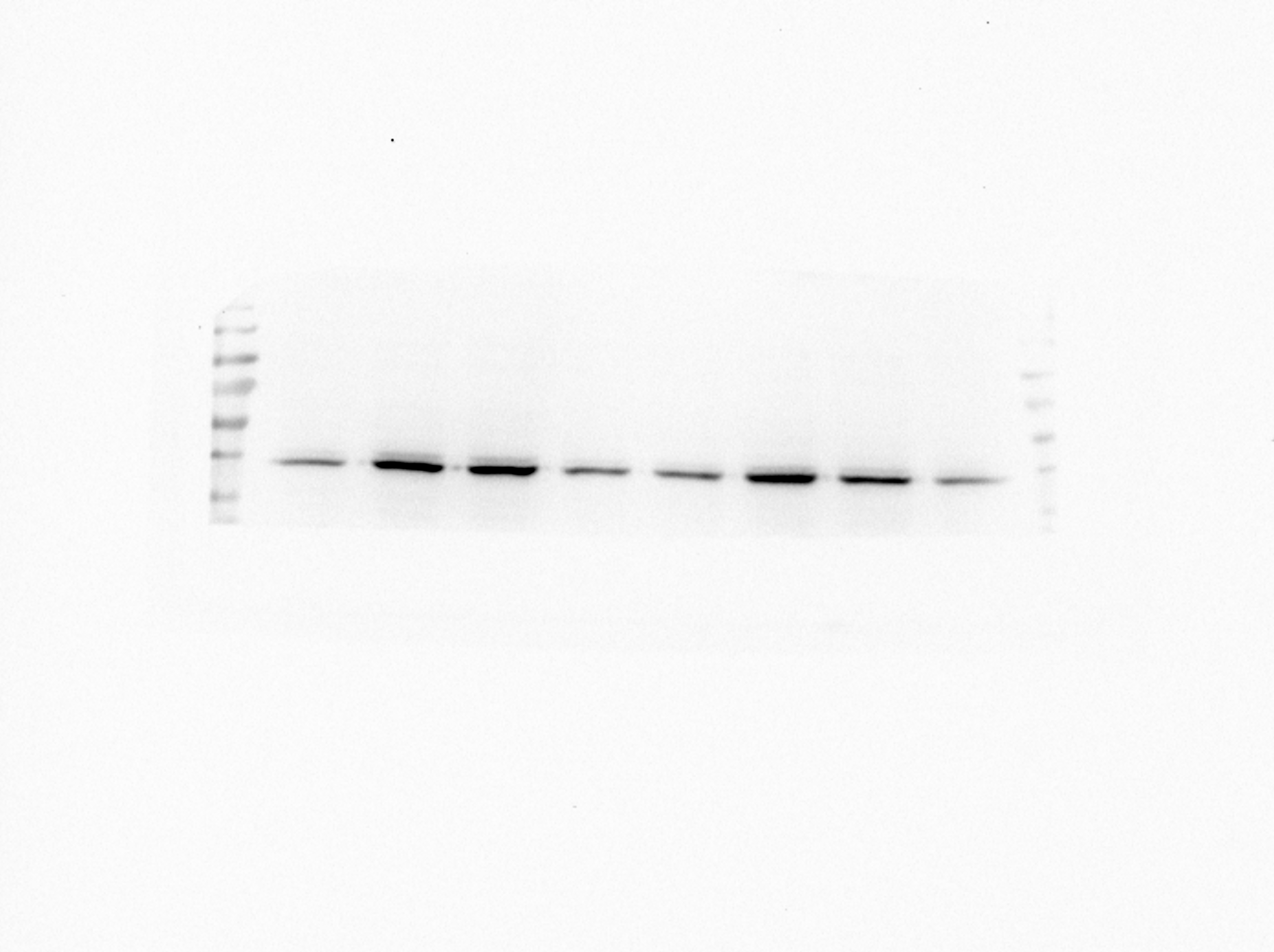

Supplement: Supplemental Information 3 [file peerj-08-10548-s003.zip › Supplemental files (Figure 5)/HaCaT/HaCaT-pERK.jpg]

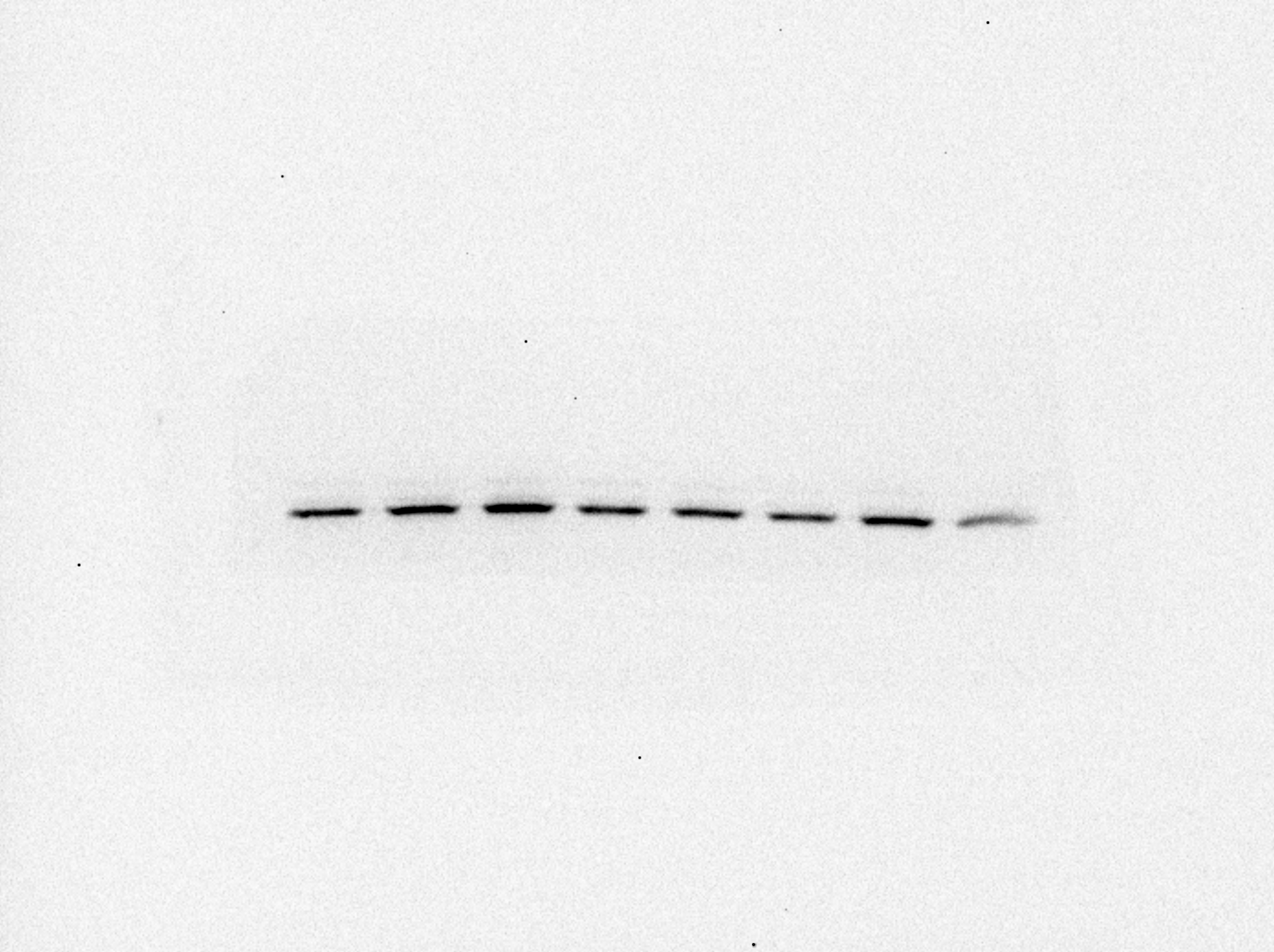

Supplement: Supplemental Information 3 [file peerj-08-10548-s003.zip › Supplemental files (Figure 5)/HaCaT/HaCaT-pp38.jpg]

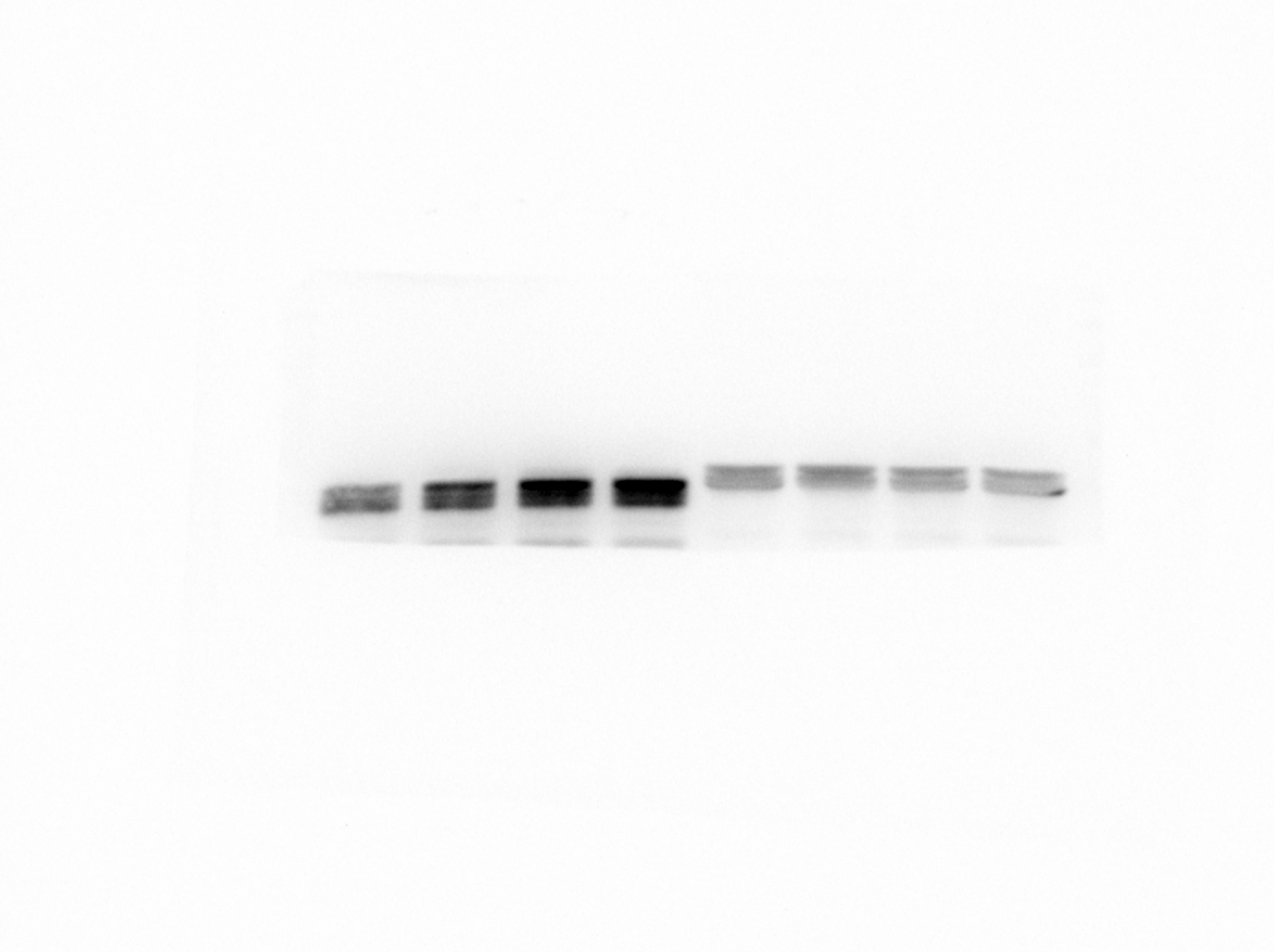

Supplement: Supplemental Information 3 [file peerj-08-10548-s003.zip › Supplemental files (Figure 5)/THP-1/THP-1-ERK.jpg]

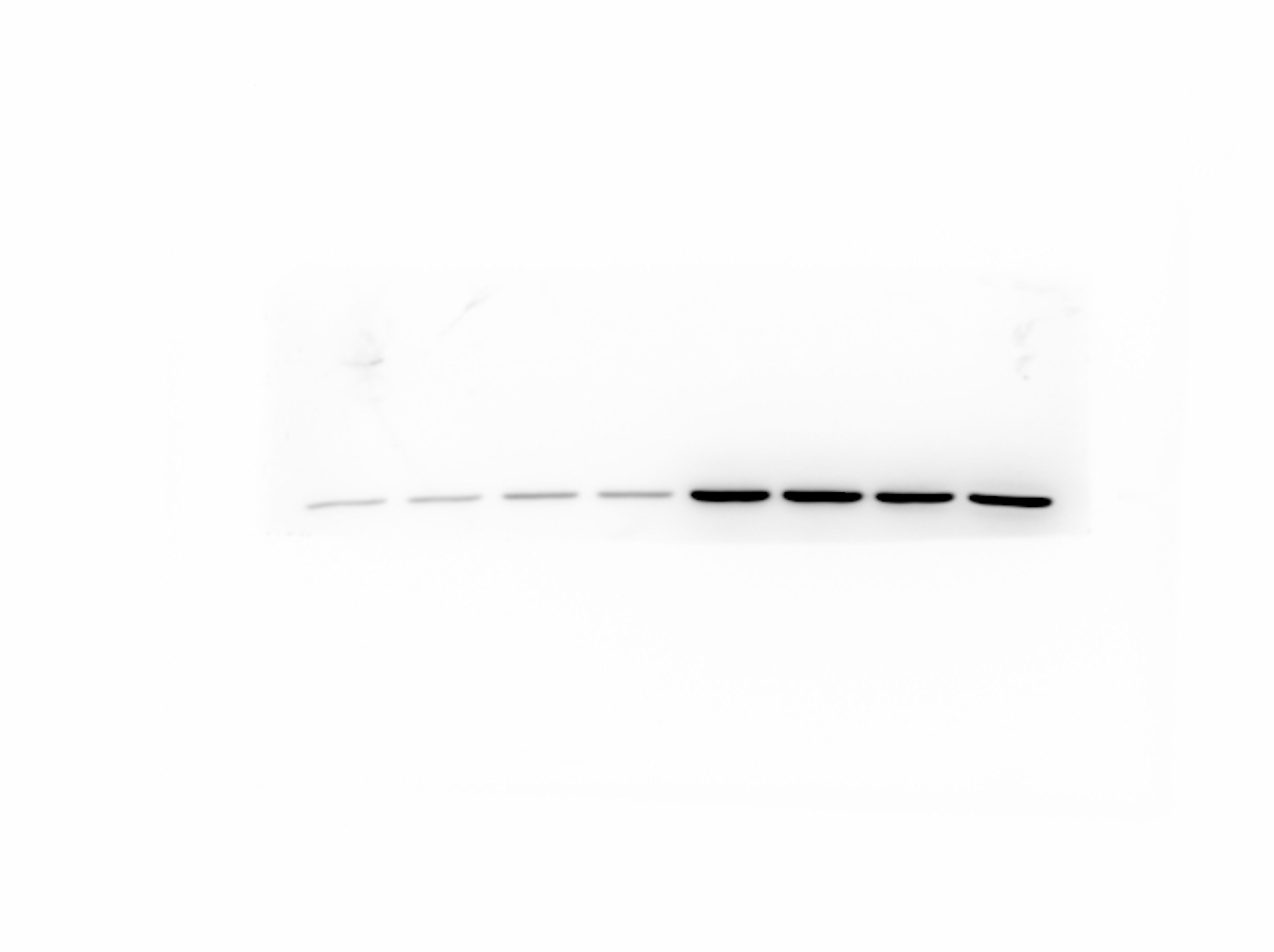

Supplement: Supplemental Information 3 [file peerj-08-10548-s003.zip › Supplemental files (Figure 5)/THP-1/THP-1-GAPDH (ERK).jpg]

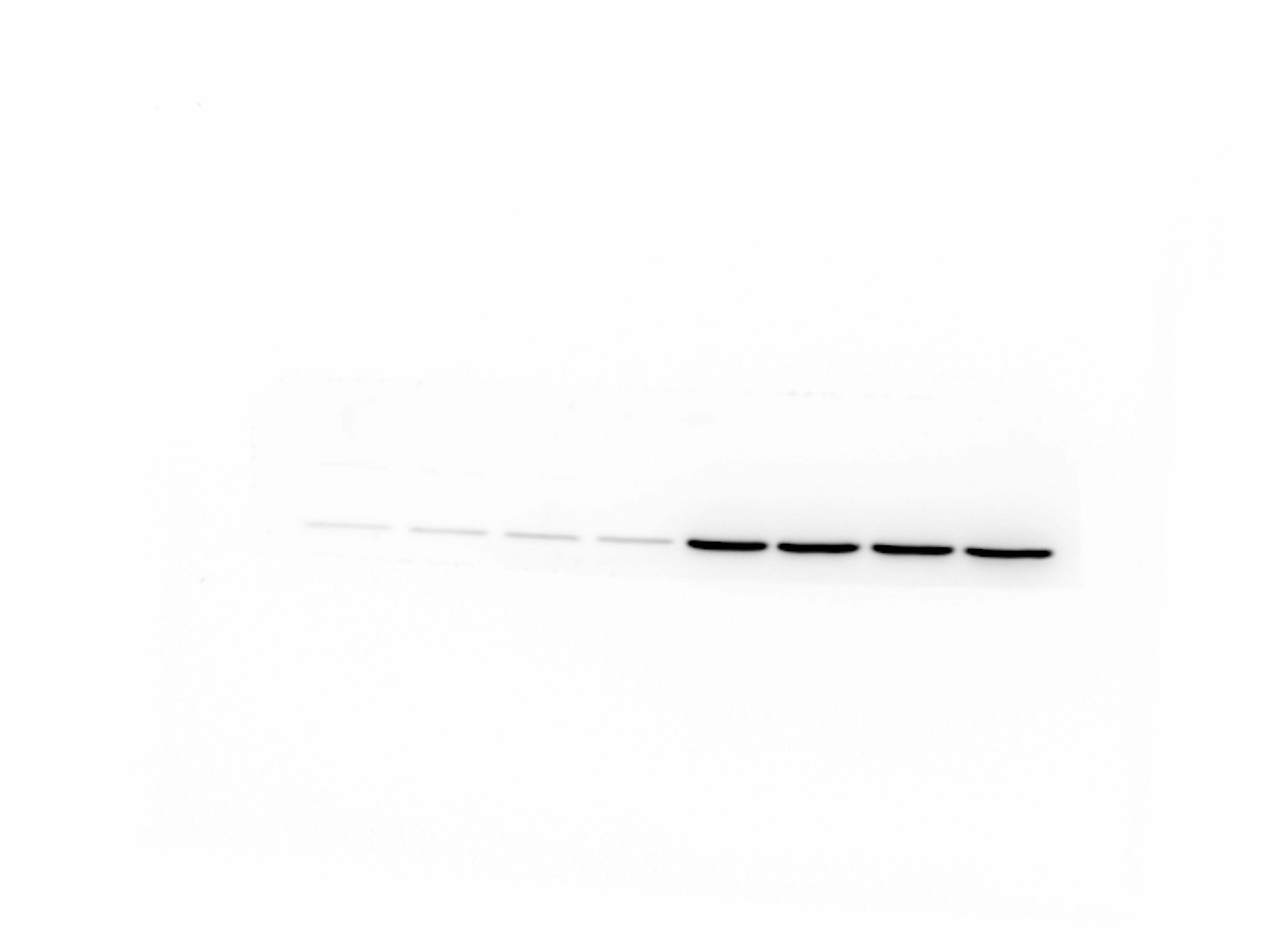

Supplement: Supplemental Information 3 [file peerj-08-10548-s003.zip › Supplemental files (Figure 5)/THP-1/THP-1-GAPDH (p38).jpg]

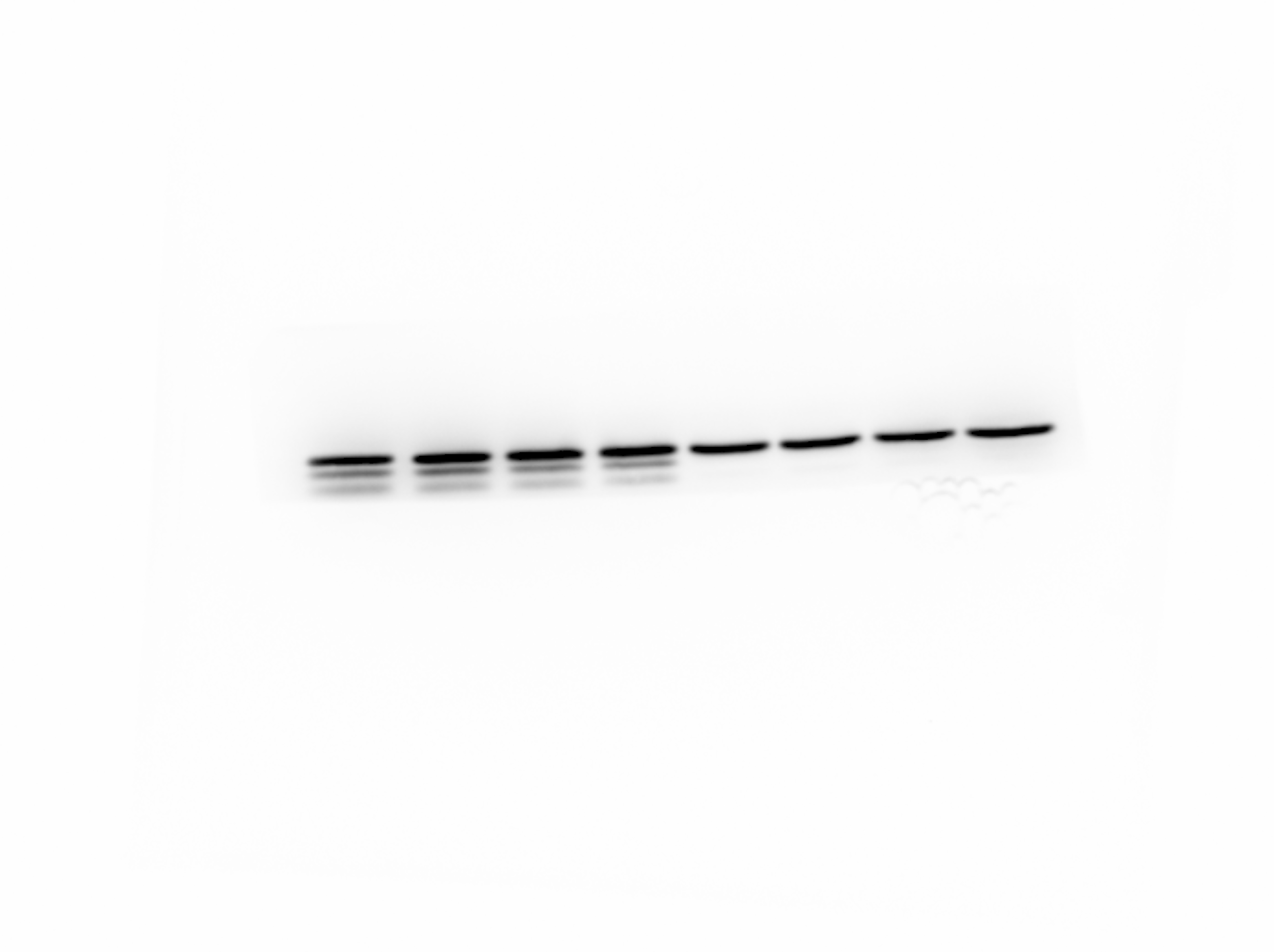

Supplement: Supplemental Information 3 [file peerj-08-10548-s003.zip › Supplemental files (Figure 5)/THP-1/THP-1-p38.jpg]

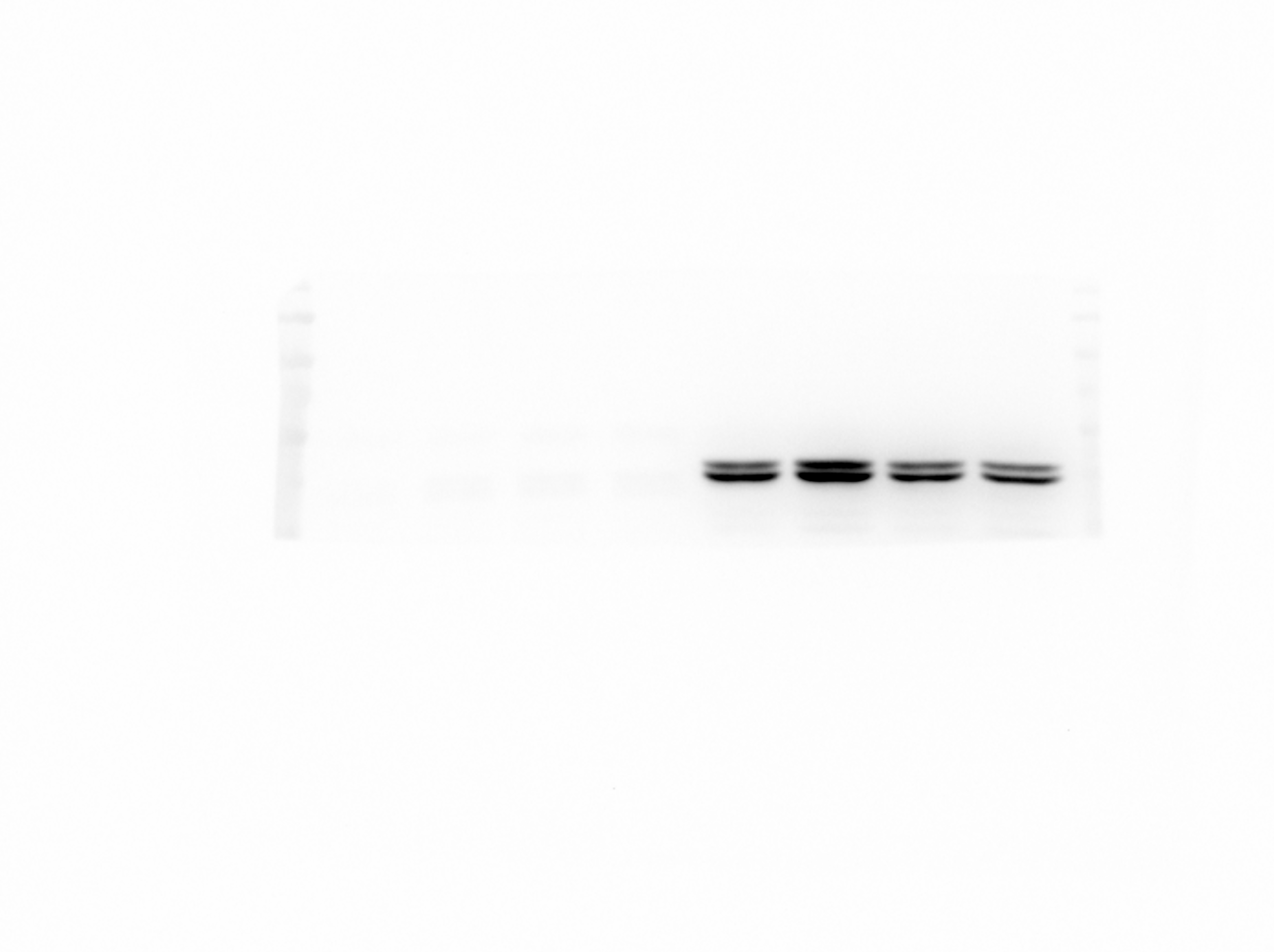

Supplement: Supplemental Information 3 [file peerj-08-10548-s003.zip › Supplemental files (Figure 5)/THP-1/THP-1-pERK.jpg]

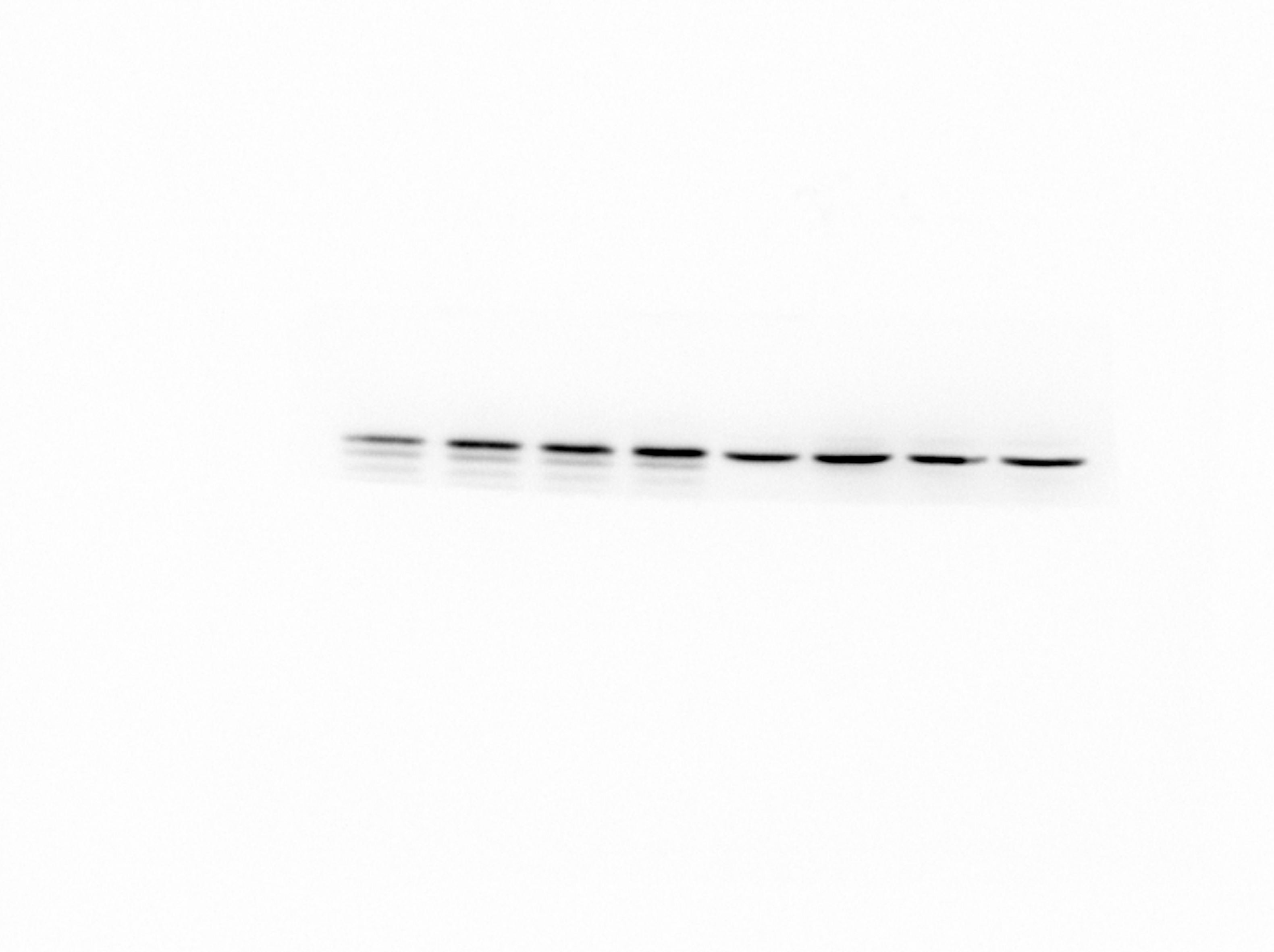

Supplement: Supplemental Information 3 [file peerj-08-10548-s003.zip › Supplemental files (Figure 5)/THP-1/THP-1-pp38.jpg]
