# Supplementary material for: Cinnamtannin B1 attenuates rosacea-like signs via inhibition of pro-inflammatory cytokine production and down-regulation of the MAPK pathway
Source: PeerJ. 2020 Dec 21;8:e10548. doi: 10.7717/peerj.10548 (PMC7759128; doi:10.7717/peerj.10548)
Supplement: Supplemental Information 3 [file peerj-08-10548-s003.zip › Supplemental files (Figure 5)/western details.pptx]

## Slide 1
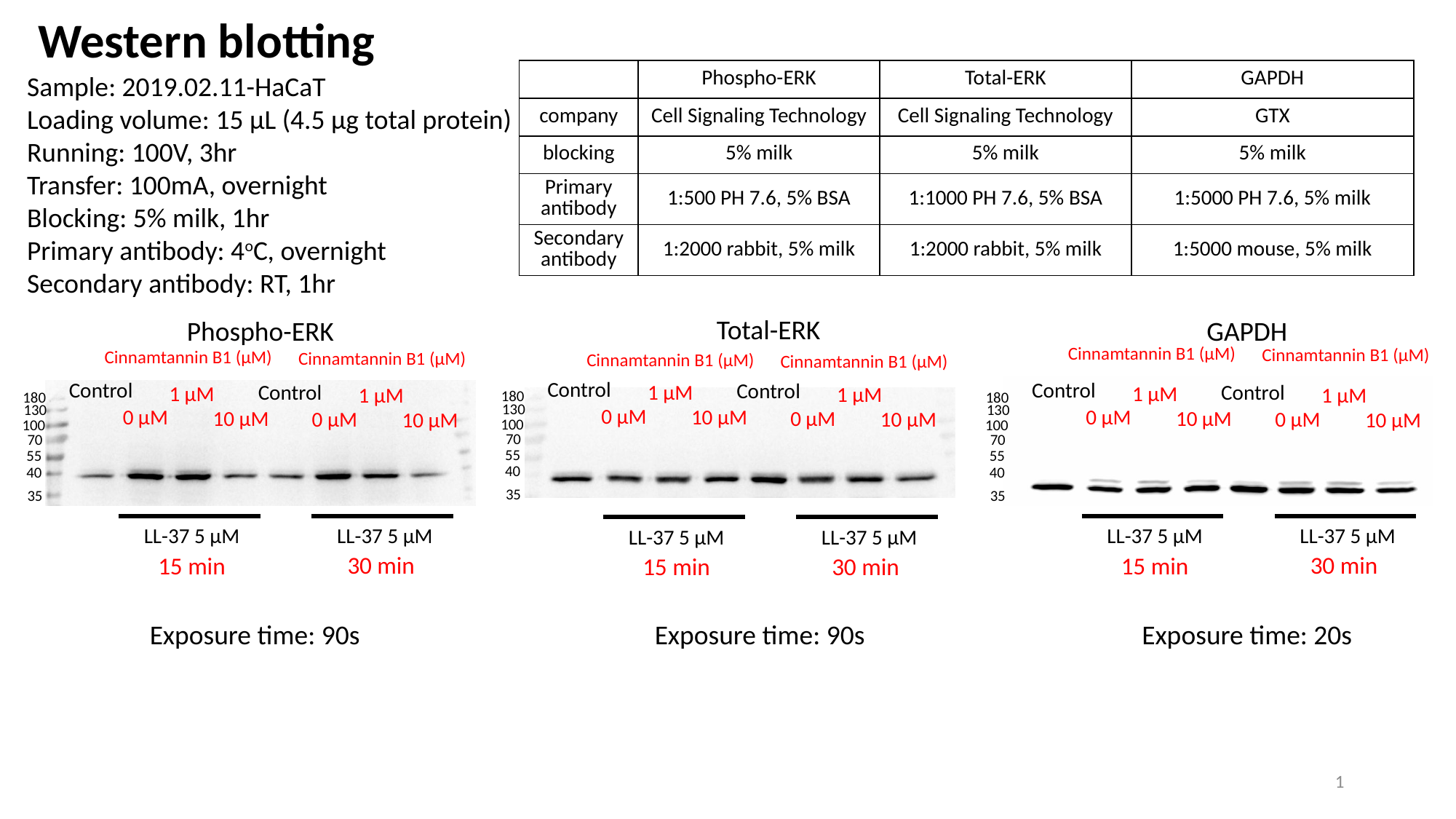

Western blotting
| | Phospho-ERK | Total-ERK | GAPDH |
| --- | --- | --- | --- |
| company | Cell Signaling Technology | Cell Signaling Technology | GTX |
| blocking | 5% milk | 5% milk | 5% milk |
| Primary antibody | 1:500 PH 7.6, 5% BSA | 1:1000 PH 7.6, 5% BSA | 1:5000 PH 7.6, 5% milk |
| Secondary antibody | 1:2000 rabbit, 5% milk | 1:2000 rabbit, 5% milk | 1:5000 mouse, 5% milk |
Sample: 2019.02.11-HaCaT
Loading volume: 15 µL (4.5 µg total protein)
Running: 100V, 3hr
Transfer: 100mA, overnight
Blocking: 5% milk, 1hr
Primary antibody: 4oC, overnight
Secondary antibody: RT, 1hr
Total-ERK
Phospho-ERK
GAPDH
Cinnamtannin B1 (µM)
Cinnamtannin B1 (µM)
Cinnamtannin B1 (µM)
Cinnamtannin B1 (µM)
Cinnamtannin B1 (µM)
Cinnamtannin B1 (µM)
Control
Control
1 µM
1 µM
180
130
0 µM
10 µM
0 µM
10 µM
100
70
55
40
35
LL-37 5 µM
LL-37 5 µM
30 min
15 min
Control
Control
1 µM
1 µM
180
130
0 µM
10 µM
0 µM
10 µM
100
70
55
40
35
LL-37 5 µM
LL-37 5 µM
30 min
15 min
Control
Control
1 µM
1 µM
180
130
0 µM
10 µM
0 µM
10 µM
100
70
55
40
35
LL-37 5 µM
LL-37 5 µM
30 min
15 min
Exposure time: 90s
Exposure time: 90s
Exposure time: 20s
1

## Slide 2
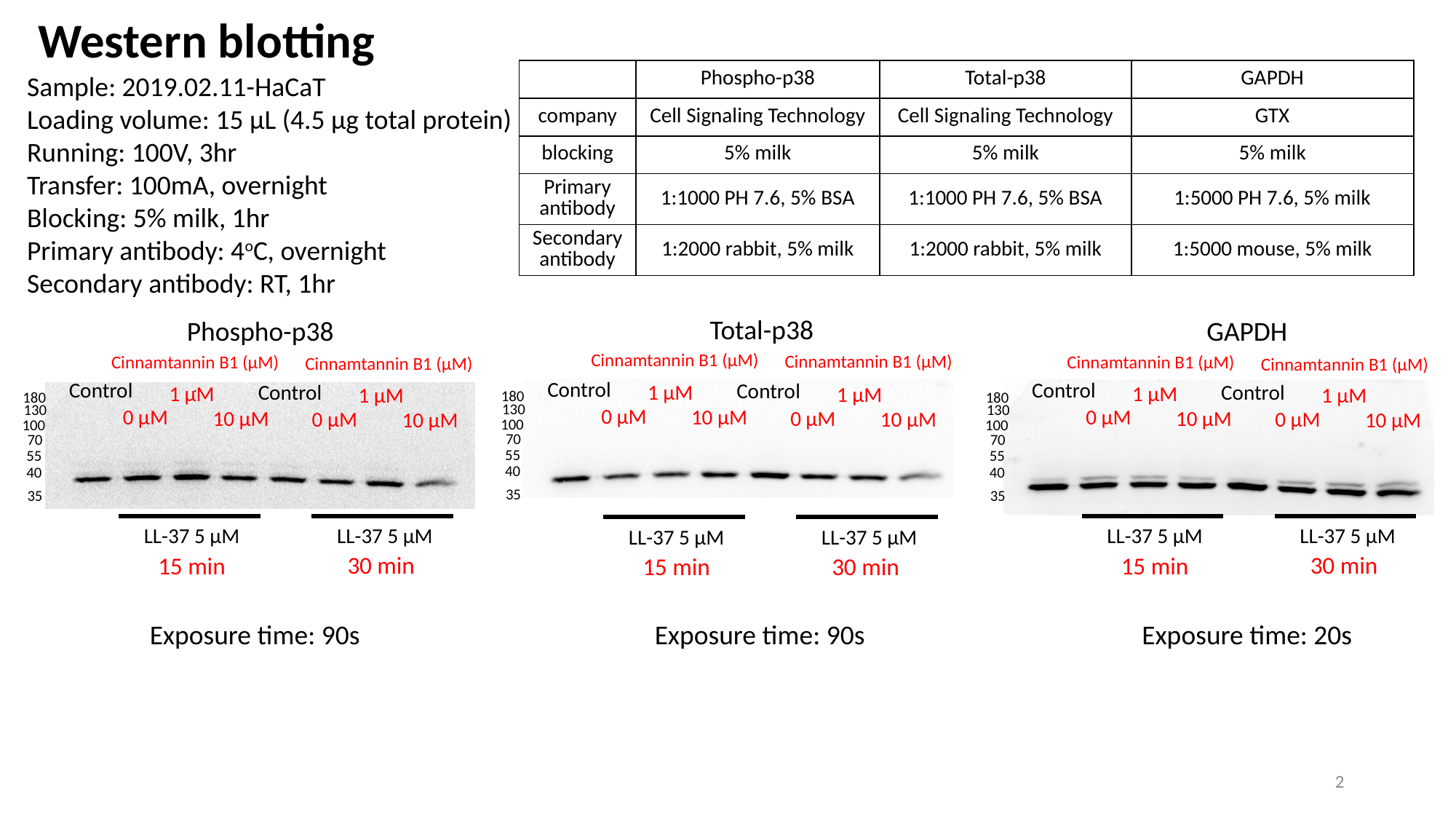

Western blotting
| | Phospho-p38 | Total-p38 | GAPDH |
| --- | --- | --- | --- |
| company | Cell Signaling Technology | Cell Signaling Technology | GTX |
| blocking | 5% milk | 5% milk | 5% milk |
| Primary antibody | 1:1000 PH 7.6, 5% BSA | 1:1000 PH 7.6, 5% BSA | 1:5000 PH 7.6, 5% milk |
| Secondary antibody | 1:2000 rabbit, 5% milk | 1:2000 rabbit, 5% milk | 1:5000 mouse, 5% milk |
Sample: 2019.02.11-HaCaT
Loading volume: 15 µL (4.5 µg total protein)
Running: 100V, 3hr
Transfer: 100mA, overnight
Blocking: 5% milk, 1hr
Primary antibody: 4oC, overnight
Secondary antibody: RT, 1hr
Total-p38
Phospho-p38
GAPDH
Cinnamtannin B1 (µM)
Cinnamtannin B1 (µM)
Cinnamtannin B1 (µM)
Cinnamtannin B1 (µM)
Cinnamtannin B1 (µM)
Cinnamtannin B1 (µM)
Control
Control
Control
Control
Control
Control
1 µM
1 µM
1 µM
1 µM
1 µM
1 µM
180
180
180
130
130
130
0 µM
10 µM
0 µM
0 µM
0 µM
10 µM
10 µM
10 µM
0 µM
0 µM
10 µM
10 µM
100
100
100
70
70
70
55
55
55
40
40
40
35
35
35
LL-37 5 µM
LL-37 5 µM
LL-37 5 µM
LL-37 5 µM
LL-37 5 µM
LL-37 5 µM
30 min
30 min
15 min
15 min
30 min
15 min
Exposure time: 90s
Exposure time: 90s
Exposure time: 20s
2

## Slide 3
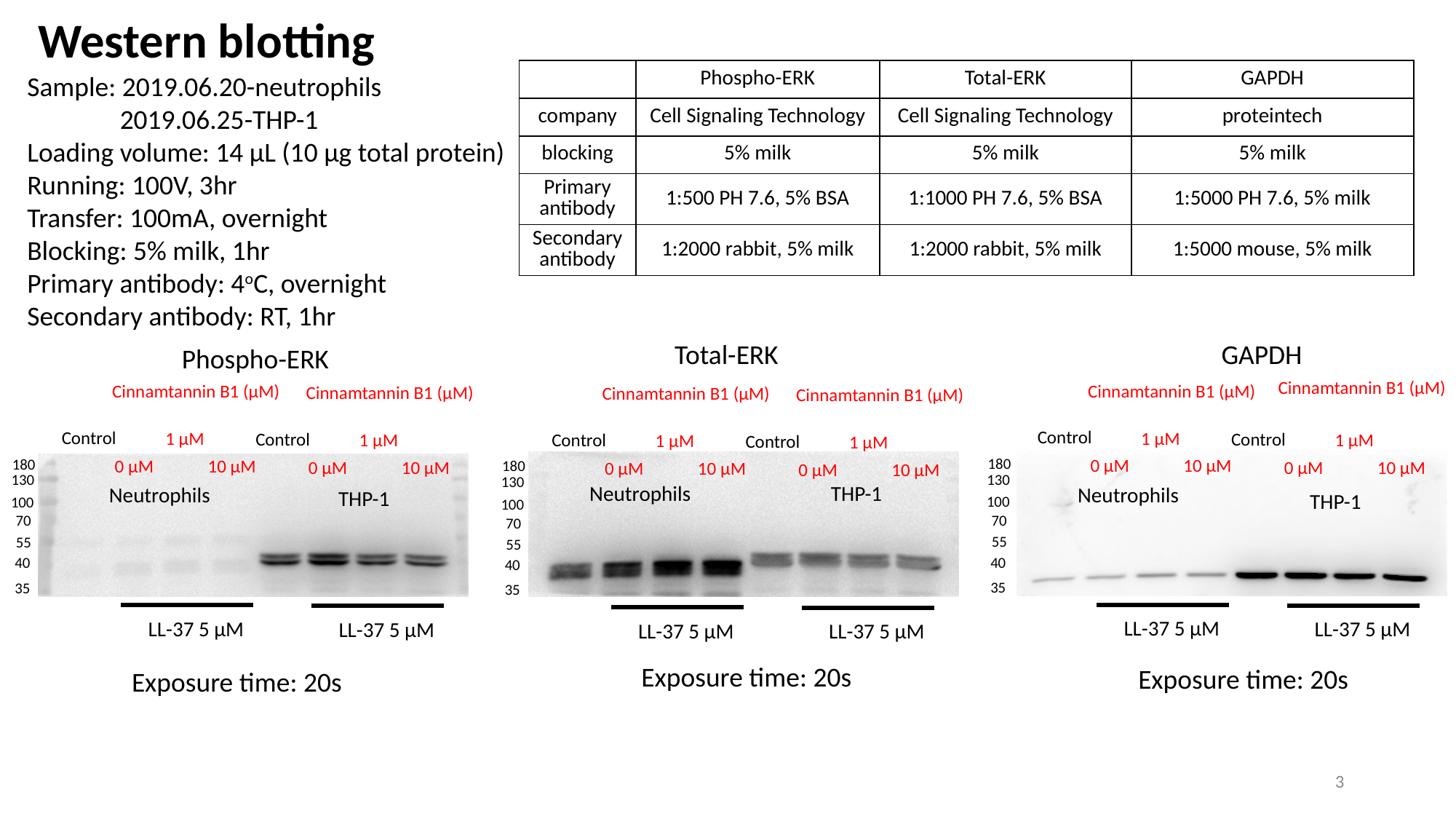

Western blotting
| | Phospho-ERK | Total-ERK | GAPDH |
| --- | --- | --- | --- |
| company | Cell Signaling Technology | Cell Signaling Technology | proteintech |
| blocking | 5% milk | 5% milk | 5% milk |
| Primary antibody | 1:500 PH 7.6, 5% BSA | 1:1000 PH 7.6, 5% BSA | 1:5000 PH 7.6, 5% milk |
| Secondary antibody | 1:2000 rabbit, 5% milk | 1:2000 rabbit, 5% milk | 1:5000 mouse, 5% milk |
Sample: 2019.06.20-neutrophils
 2019.06.25-THP-1
Loading volume: 14 µL (10 µg total protein)
Running: 100V, 3hr
Transfer: 100mA, overnight
Blocking: 5% milk, 1hr
Primary antibody: 4oC, overnight
Secondary antibody: RT, 1hr
GAPDH
Total-ERK
Phospho-ERK
Cinnamtannin B1 (µM)
Cinnamtannin B1 (µM)
Cinnamtannin B1 (µM)
Cinnamtannin B1 (µM)
Cinnamtannin B1 (µM)
Cinnamtannin B1 (µM)
Control
Control
1 µM
1 µM
Control
Control
Control
1 µM
1 µM
1 µM
Control
1 µM
180
0 µM
10 µM
180
0 µM
10 µM
0 µM
10 µM
0 µM
10 µM
180
0 µM
10 µM
0 µM
10 µM
130
130
130
Neutrophils
THP-1
Neutrophils
Neutrophils
THP-1
THP-1
100
100
100
70
70
70
55
55
55
40
40
40
35
35
35
LL-37 5 µM
LL-37 5 µM
LL-37 5 µM
LL-37 5 µM
LL-37 5 µM
LL-37 5 µM
Exposure time: 20s
Exposure time: 20s
Exposure time: 20s
3

## Slide 4
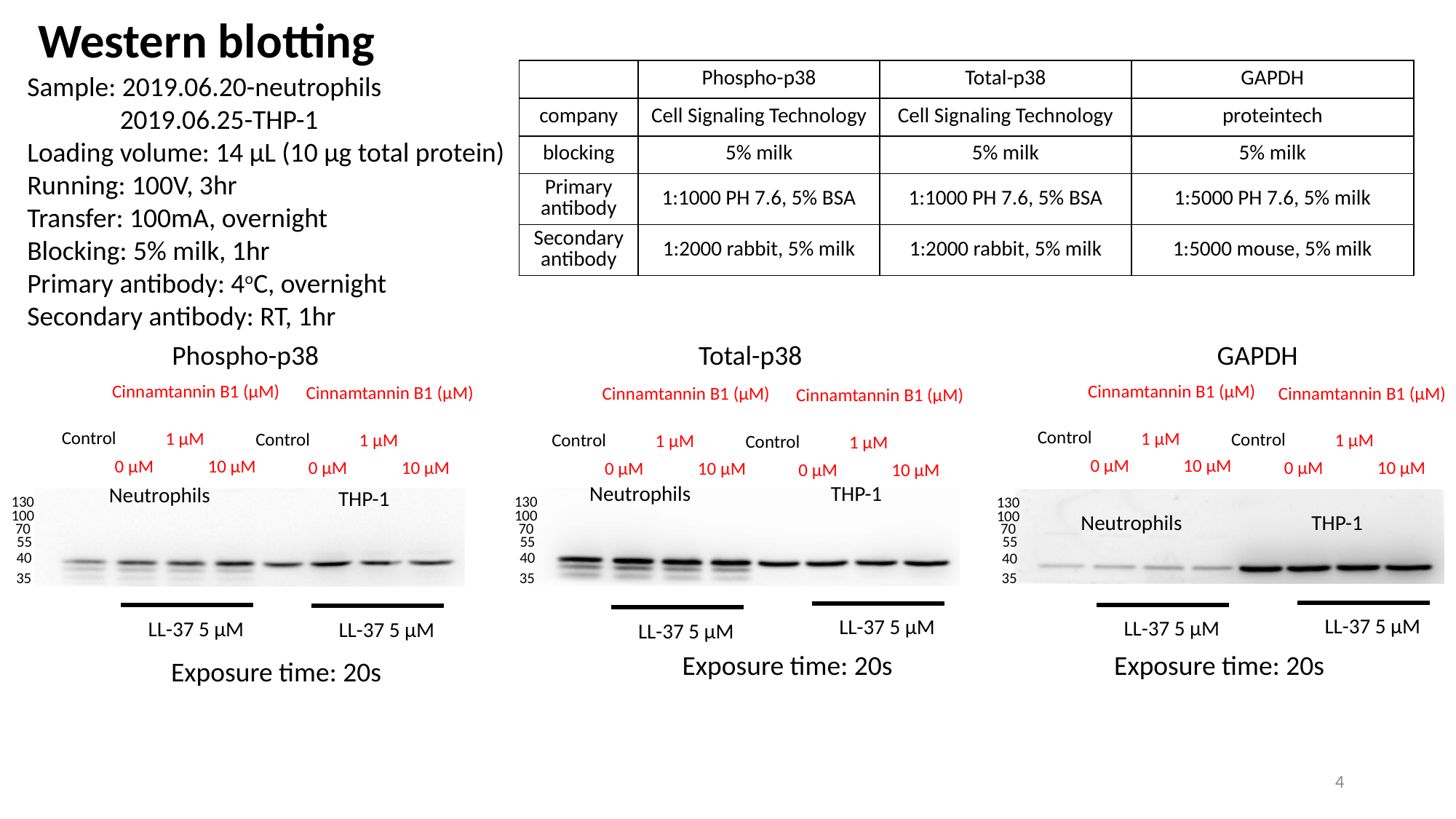

Western blotting
| | Phospho-p38 | Total-p38 | GAPDH |
| --- | --- | --- | --- |
| company | Cell Signaling Technology | Cell Signaling Technology | proteintech |
| blocking | 5% milk | 5% milk | 5% milk |
| Primary antibody | 1:1000 PH 7.6, 5% BSA | 1:1000 PH 7.6, 5% BSA | 1:5000 PH 7.6, 5% milk |
| Secondary antibody | 1:2000 rabbit, 5% milk | 1:2000 rabbit, 5% milk | 1:5000 mouse, 5% milk |
Sample: 2019.06.20-neutrophils
 2019.06.25-THP-1
Loading volume: 14 µL (10 µg total protein)
Running: 100V, 3hr
Transfer: 100mA, overnight
Blocking: 5% milk, 1hr
Primary antibody: 4oC, overnight
Secondary antibody: RT, 1hr
Phospho-p38
GAPDH
Total-p38
Cinnamtannin B1 (µM)
Cinnamtannin B1 (µM)
Cinnamtannin B1 (µM)
Cinnamtannin B1 (µM)
Cinnamtannin B1 (µM)
Cinnamtannin B1 (µM)
Control
Control
1 µM
1 µM
Control
Control
Control
1 µM
1 µM
1 µM
Control
1 µM
0 µM
10 µM
0 µM
10 µM
0 µM
10 µM
0 µM
10 µM
0 µM
10 µM
0 µM
10 µM
Neutrophils
THP-1
Neutrophils
THP-1
130
130
130
100
100
100
THP-1
Neutrophils
70
70
70
55
55
55
40
40
40
35
35
35
LL-37 5 µM
LL-37 5 µM
LL-37 5 µM
LL-37 5 µM
LL-37 5 µM
LL-37 5 µM
Exposure time: 20s
Exposure time: 20s
Exposure time: 20s
4
